# *In Vitro* and *In Silico* Evaluation of Anticholinesterase and Antidiabetic Effects of Furanolabdanes and Other Constituents from *Graptophyllum pictum* (Linn.) Griffith

**DOI:** 10.3390/molecules28124802

**Published:** 2023-06-16

**Authors:** Nathalie Tanko Metiefeng, Alfred Ngenge Tamfu, Maurice Fotsing Tagatsing, Turibio Kuiate Tabopda, Selcuk Kucukaydin, Martin Noah Mbane, Alex de Theodore Atchade, Emmanuel Talla, Celine Henoumont, Sophie Laurent, El Hassane Anouar, Rodica Mihaela Dinica

**Affiliations:** 1 Department of Organic Chemistry, Faculty of Science, The University of Yaounde I, Yaounde 812, Cameroon; 2 Department of Chemical Engineering, School of Chemical Engineering and Mineral Industries, University of Ngaoundere, Ngaoundere 454, Cameroon; 3 Department of Medical Services and Techniques, Koycegiz Vocational School of Health Services, Mugla Sitki Kocman University, Mugla 48800, Turkey; 4 Department of Chemistry, Physics and Environment, Faculty of Sciences and Environment, ‘Dunarea de Jos’ University of Galati, 47 Domneasca Str., 800008 Galati, Romania; 5 Laboratory of NMR and Molecular Imaging, Department of General, Organic and Biomedical Chemistry, University of Mons, B-7000 Mons, Belgium; 6 Department of Chemistry, College of Sciences and Humanities in Al-Kharj, Prince Sattam bin Abdulaziz University, Al-Kharj 11942, Saudi Arabia

**Keywords:** *Graptophyllum pictum*, furanolabdanes, anticholinesterase, α-amylase, α-glucosidase, molecular docking

## Abstract

*Graptophyllum pictum* is a tropical plant noticeable for its variegated leaves and exploited for various medicinal purposes. In this study, seven compounds, including three furanolabdane diterpenoids, i.e., Hypopurin E, Hypopurin A and Hypopurin B, as well as with Lupeol, β-sitosterol 3-*O*-β-d-glucopyranoside, stigmasterol 3-*O*-β-d-glucopyranoside and a mixture of β-sitosterol and stigmasterol, were isolated from *G. pictum*, and their structures were deduced from ESI-TOF-MS, HR-ESI-TOF-MS, 1D and 2D NMR experiments. The compounds were evaluated for their anticholinesterase activities against acetylcholinesterase (AChE) and butyrylcholinesterase (BchE), as well as their antidiabetic potential through inhibition of α-glucosidase and α-amylase. For AChE inhibition, no sample had IC50 within tested concentrations, though the most potent was Hypopurin A, which had a percentage inhibition of 40.18 ± 0.75%, compared to 85.91 ± 0.58% for galantamine, at 100 µg/mL. BChE was more susceptible to the leaves extract (IC_50_ = 58.21 ± 0.65 µg/mL), stem extract (IC_50_ = 67.05 ± 0.82 µg/mL), Hypopurin A (IC_50_ = 58.00 ± 0.90 µg/mL), Hypopurin B (IC_50_ = 67.05 ± 0.92 µg/mL) and Hypopurin E (IC_50_ = 86.90 ± 0.76 µg/mL). In the antidiabetic assay, the furanolabdane diterpenoids, lupeol and the extracts had moderate to good activities. Against α-glucosidase, lupeol, Hypopurin E, Hypopurin A and Hypopurin B had appreciable activities but the leaves (IC_50_ = 48.90 ± 0.17 µg/mL) and stem (IC_50_ = 45.61 ± 0.56 µg/mL) extracts were more active than the pure compounds. In the α-amylase assay, stem extract (IC_50_ = 64.47 ± 0.78 µg/mL), Hypopurin A (IC_50_ = 60.68 ± 0.55 µg/mL) and Hypopurin B (IC_50_ = 69.51 ± 1.30 µg/mL) had moderate activities compared to the standard acarbose (IC_50_ = 32.25 ± 0.36 µg/mL). Molecular docking was performed to determine the binding modes and free binding energies of Hypopurin E, Hypopurin A and Hypopurin B in relation to the enzymes and decipher the structure–activity relationship. The results indicated that *G. pictum* and its compounds could, in general, be used in the development of therapies for Alzheimer’s disease and diabetes.

## 1. Introduction

Oxidative stress is responsible for numerous human ailments. It occurs as a result of an imbalance between generation of oxidant species and antioxidants in living systems, and often spreads to other cell targets and tissues [1,2,3]. This unfavorable situation involving excessive generation of reactive nitrogen species (RNS) and reactive oxygen species (ROS), such as hydroxyl radicals, superoxide anion, nitric oxide and peroxides, leads to undesirable effects and damage to molecules such as lipids, proteins and DNA [4]. This oxidative stress is usually involved in the onset of hypertension, obesity enzymatic disorders, such as Alzheimer’s disease (AD), and diabetes [2,5,6]. Besides oxidative stress, there are multiple molecular mechanisms that are possibly involved in neurodegeneration and type 2 diabetes, amongst which are inflammation, mitochondrial dysfunction, endoplasmic reticulum stress and autophagy [7]. Insulin resistance is becoming more and more evident as the most common feature that links both type 2 diabetes and AD, having evident pathophysiological homologies and molecular pathways [8]. Obesity and diabetes are risk factors for Alzheimer’s disease.

Alzheimer’s disease (AD) is a prevalent neurodegenerative ailment, which is characterized by irreversible and progressive loss of memory, emotional dysfunction, decreased cognitive abilities, impairment, dementia and, ultimately, death of patients [9,10]. The number of people living with AD in the world is estimated at about 50 million, and this figure is expected to triple to around 152 million by 2050 [11,12]. The global number of AD cases and mortalities are increasing: from 1990 to 2019, the number of cases of Alzheimer’s disease and other dementia-related conditions rose by almost 147.95% and 160.84%, respectively [13]. A great number of studies suggest that AD may be related to many hypotheses, including the beta-amyloid (Aβ), cholinergic, tau and neuroinflammatory hypotheses [14]. As AD is often associated with cholinergic deficiency, cholinesterase inhibitors can help to boast acetylcholine concentrations and increase nerve transmission, which is a suitable strategy to relieve symptoms of dementia [15]. Many cholinesterase inhibitors from naturally derived and synthetic sources were previously developed as suitable remedies for AD, with greater efficacy and low toxicity attributed to natural remedies [16,17]. This search is ongoing due to cases of side effects being previously reported for some of the available drugs, as well as the fact that these treatments have not stopped the progression of the disease completely.

Diabetes mellitus (DM) is a fast-growing disorder that results from carbohydrate and fat metabolism, and is associated with chronic hyperglycemia and characterized by high amounts of sugar in the blood resulting from the deficiency and/or activity of insulin, which is the pancreatic hormone which regulates glycaemia [18,19]. Type 1 diabetes mellitus can be caused by insulin production deficiency, while Type 2 diabetes mellitus results from the insufficiency or inefficacy of insulin produced [19]. The global prevalence of diabetes cases was estimated at 9.3% (463 million people) in 2019, and is expected to rise to 10.2% (578 million) by 2030 and 10.9% (700 million) by 2045 [20]. Certain drugs, including miglitol, voglibose, acarbose and pycnogenol, are usually used to manage hyperglycemia, though some undesirable side effects, such as diarrhea, abdominal pain, flatulence, bloating and discomfort, have been observed [21]. This issue motivates the ongoing research into natural antidiabetic compounds, such as phenolics, terpenoids, flavonoids, alkaloids and coumarins from medicinal plants [22,23,24]. The inhibition of carbohydrate digestive enzymes (α-amylase and α-glucosidase) is a good means of reducing blood glucose levels to normal, and various phytochemicals and several synthetic compounds were previously applied to this effect, especially those that are able to restrict or prevent glycoside and starch hydrolysis [25,26,27,28]. The principal source of hypoglycemic agents is natural products, which are highly cherished due to their low cost and availability relative to the high cost and side effects from some conventional drugs [29]. 

*Graptophyllum pictum* is a popular medicinal shrub of the Acanthaceae family that is popularly known as ‘Joseph’s coat’ or the ‘caricature plant’ because of its variegated colored leaves; the plant grows in various tropical regions, including the Pacific regions and Western and Central Africa [30,31]. *Graptophyllum pictum* is widely used for wound healing, constipation, earache, sores, swellings, hemorrhoid, ear diseases, antipyretic, scabies, urinary infection, smoothing skins wounds, hepatomegaly, laxative, diuretic analgesics, menstrual problems, treat tonsillitis, abscess, ulcers and rheumatism [32,33,34]. Scientific studies reported pharmacological benefits, such as anti-haemorrhoid, antidiabetic, uterotonic, antimicrobial, anti-inflammatory, antioxidant, estrogenic, analgesic, abortifacient, hypoglycemic, anti-hemorrhoid and wound healing properties [30,31,35,36]. Previous chemical investigations and phytochemical screening of *Graptophyllum pictum* reports the presence triterpenes, flavonoids, steroids, tannins and saponins [35,36]. 

This work aimed to contribute to the search for new antidiabetic and anticholinesterase phytochemicals. This study involves the isolation and characterization of compounds from *Graptophyllum pictum*, as well as the evaluation of their α-amylase, α-glucosidase and cholinesterase inhibitory potential.

## 2. Results

### 2.1. Characterization of Isolated Compounds

**Figure 1:**
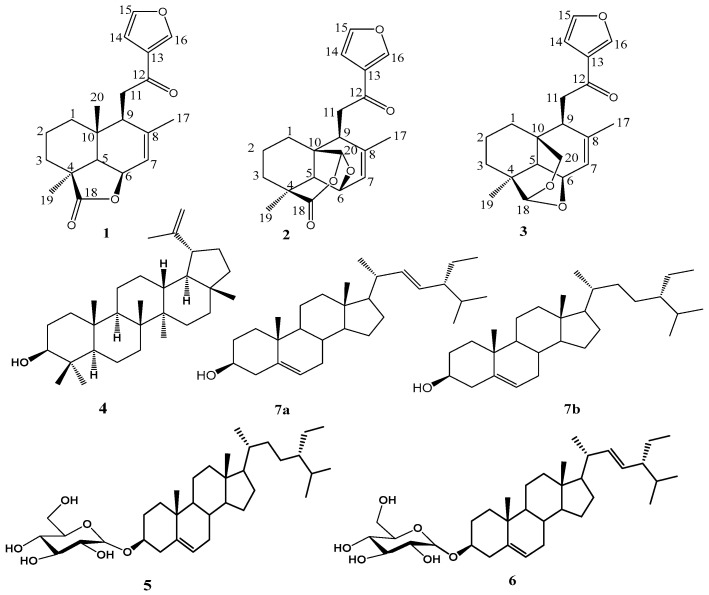
Chemical structures of compounds isolated from *G. pictum*.

The chemical structures of the phytochemicals isolated from the leaves and stems of *G. pictum* are provided in Figure 1. Seven compounds were isolated using column chromatography, and their structures were determined from NMR and ESI-TOF-MS data and classified as follows: three furanolabdane diterpenoids, i.e., Hypopurin E, Hypopurin A and Hypopurin B [37], as well as with Lupeol [38], a mixture of stigmasterol and β-sitosterol [39], β-sitosterol 3-*O*-β-d-glucopyranoside [40] and stigmasterol 3-*O*-β-d-glucopyranoside [41]. 

Compound **3** was obtained from the stems extract of *Graptophyllum pictum*, being a white amorphous powder in the eluent hexane:ethylacetate (80:20). The molecular formula of this compound was precisely deduced from the ESI-TOF-MS data in positive mode from the quasi-molecular ion [M + H]^+^ at *m*/*z* 329.7 g/mol for C_20_H_25_O_4_, from which findings the molecular formula C_20_H_24_O_4_ was deduced. The high resolution ESI-TOF-MS spectrum of compound **3** (Figure S2) showed a pseudo-molecular ion peak at [M + Na]^+^ at *m/z* 351.1572, which corresponded to the molecular formula C_20_H_24_O_4_Na (C_20_H_24_O_4_ + Na, *m/z* 351.1572), suggesting the existence of nine double bond equivalences. The ^13^C NMR broadband spectrum of this compound indicated 20 carbon signals, suggesting that it was a diterpenoid. The ^13^C NMR in DEPT mode indicated signals of two methyl, five methylene, eight methine and five quaternary carbon atoms. The signal of a carbonyl group attributable to the conjugated ketone carbon atom C-12 was observed at *δ*_C_ 194.4 ppm. Three characteristic proton signals at *δ_H_* 8.54 (H-15), 7.70 (H-15) and 6.85 ppm (H-14) were observed and exhibited HSQC correlation with carbon signals at *δ_C_* 149.1 (C-16), 145.5 (H-15) and 109.3 (H-14), respectively; this set of information suggests the presence of a three-substituted furan ring in compound 3, which conforms with the reported data [37,42]. The signal of a quaternary carbon at *δ_C_* 128.4 ppm (C-13) was present and exhibited ^2^J correlations with the protons at *δ_H_* 8.54 (H-16) and 6.85 ppm (H-14), as well as a ^3^J correlation with the proton at *δ_H_* 7.70 ppm in the HMBC spectrum. Additionally, the protons with signals at *δ_H_* 2.99 (H-11) ppm exhibited HMBC correlation peaks in line with the carbon atoms with signals at *δ_C_* 141.6 (C-8) and *δ_C_* 194.4 (C-12). The oxymethylene carbon at *δ_C_* 65.4 was proven to bear the proton with signal *δ_H_* 4.79 (H-20), and this proton (H-20) showed characteristic ^3^J HMBC correlations in line with the carbon with signal *δ_C_* 108.0 ppm (C-18), which, in turn, had ^3^J HMBC correlations with the protons (3H) with singlet signals *δ_H_* 0.96 (H-19). Sets of HMBC cross-peaks between H-19 and C-3, C-4, C-5, and C-20 and H-17 and C-8, C-7, and C-9 were observed, indicating octahydronaphthalene moiety [37]. The HMBC data also showed cross-peaks between H-11 and C-8, C-9, C-10, and C-12 and H-14 and C-12, C-13, C-15, and C-16, suggesting that a 2-(3-furanyl)-2-oxoethyl group was linked to C-8 [37]. The COSY spectrum had cross-peaks between H-6 (*δ_H_* 4.37 ppm) and two protons and H-5 (*δ_H_* 1.63 ppm) and H-7 (*δ_H_* 5.76 ppm), which revealed that an oxymethine group was attached to a double bond and a methine group. The HSQC and the COSY spectra were used to establish the linkages at C-1, C-2, C-3, C-5, C-6, C-7, C-9, C-11, C-18 and C-20. It was previously explained that the NOESY of such a compound exhibited cross-peaks of H-5/H-6, H-9, H-6/H-5, H-19, and H-20/H-11, implying that H-5, H-6 and H-19 are located on the α-face of the octahydronaphthalene ring, while the 2-(3-furanyl)-2-oxoethyl and methyl groups at C-9 and C-10 are located on the *β*-face [37]. From the preceding elucidation of spectral data, the structure of diterpene compound **3** was established and given the name **Hypopurin E**.

### 2.2. Anticholinesterase and Antidiabetic Activities

**Figure 2:**
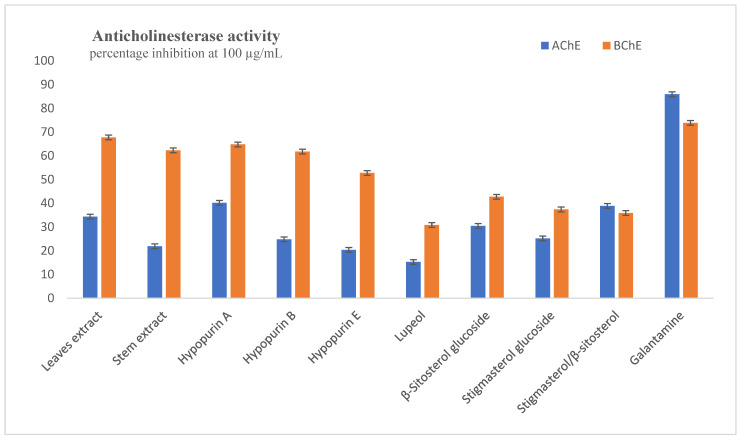
Inhibition of acetylcholinesterase and butyrylcholinesterase at 100 µg/mL.

AChE and BChE are two enzymes that are responsible for lowering the amounts of acetylcholine necessary for nerve transmission, thus increasing acetylcholine levels. The inhibitory potential of extracts and compounds on both enzymes were evaluated, and percentage inhibitions at the highest test concentration of 100 µg/mL are plotted in Figure 2. The most active sample acting against AChE was Hypopurin A, with percentage inhibition being 40.18 ± 0.75% at 100 µg/mL; this result was low compared to galantamine, which had 85.91 ± 0.58% inhibition at same concentration. The other two furanolabdanes, i.e., Hypopurin B and Hypopurin E, had percentage inhibitions of 24.80 ± 0.33% and 20.37 ± 0.56%, respectively, while the two sterol glucosides, i.e., β-Sitosterol glucoside and Stigmasterol glucoside, exhibited 30.45 ± 0.23% and 25.20 ± 0.94%, respectively. The leaves and stem extracts inhibited AChE at 100 µg/mL of 34.38 ± 0.51% and 21.87 ± 0.48%, respectively. No sample had IC_50_ values for AChE within the test concentration. Against BChE, the activities were good, especially for the furanolabdanes and the extracts. The percentage inhibitions were good for leaves extract (67.74 ± 0.80%), stem extract (62.30 ± 1.04%), Hypopurin A (64.78 ± 0.93%), Hypopurin B (61.76 ± 1.15%) and Hypopurin E (52.77 ± 1.07%) relative to galantamine (73.85 ± 0.30%). The IC_50_ values shown in Table 1 were exhibited within tested concentrations for leaf extracts (IC_50_ = 58.21 ± 0.65 µg/mL), stem extracts (IC_50_ = 67.05 ± 0.82 µg/mL), Hypopurin A (IC_50_ = 58.00 ± 0.90 µg/mL), Hypopurin B (IC_50_ = 67.05 ± 0.92 µg/mL) and Hypopurin E (IC_50_ = 86.90 ± 0.76 µg/mL).

**Figure 3:**
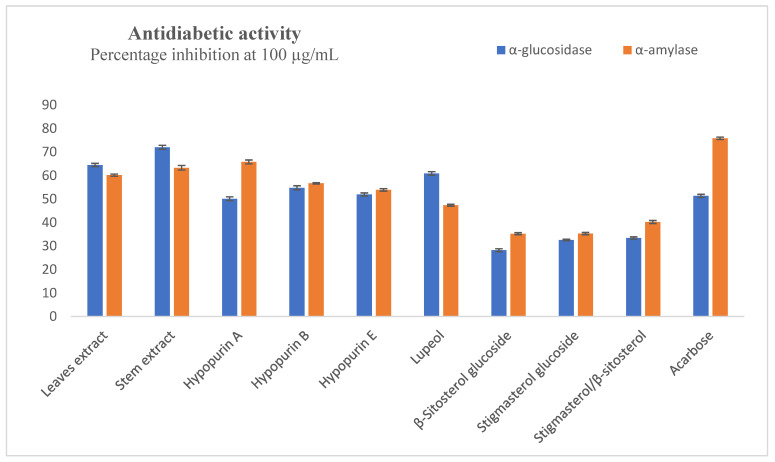
Inhibition of α-amylase and α-glucosidase at 100 µg/mL.

The inhibition of α-amylase and α-glucosidase can greatly delay the hydrolysis of starch into sugars and reduce the blood glucose level. Inhibitors of these enzymes have applications as antidiabetic agents. The percentage inhibitory effects of the compounds and extracts against these enzymes at the highest test concentration of 100 µg/mL are presented in Figure 3. Lupeol was the most active compound in the α-glucosidase assays with 60.91 ± 0.76% inhibition, while there were percentage inhibitions of 54.79 ± 0.85%, 51.95 ± 0.63% and 50.13 ± 0.81% for Hypopurin E, Hypopurin B and Hypopurin A, respectively. The standard acarbose had 51.35 ± 0.65% inhibition, while the leaf and stem extracts showed percentage inhibitions of 64.49 ± 0.69% and 72.04 ± 0.83%, respectively. From the IC_50_ values presented in Table 1, the leaf (IC_50_ = 48.90 ± 0.17 µg/mL) and stem (IC_50_ = 45.61 ± 0.56 µg/mL) extracts were more active than the pure compounds. In the α-amylase assay, percentage inhibitions were good, being 100 µg/mL for leaf extract (60.17 ± 0.47%), stem extract (63.30 ± 0.98%), Hypopurin A (65.79 ± 0.84%), Hypopurin B (56.68 ± 0.24%) and Hypopurin C (53.90 ± 0.52%). The other compounds could not inhibit up to 50% at the concentration of 100 µg/mL, while acarbose had 75.84 ± 0.52% inhibition at this concentration. From the determined IC_50_ values presented in Table 1, the stem extract (IC_50_ = 64.47 ± 0.78 µg/mL), Hypopurin A (IC_50_ = 60.68 ± 0.55 µg/mL) and Hypopurin B (IC_50_ = 69.51 ± 1.30 µg/mL) exhibited relatively moderate activities compared to the standard acarbose (IC_50_ = 32.25 ± 0.36 µg/mL). 

### 2.3. Molecular Docking Studies

**Figure 4:**
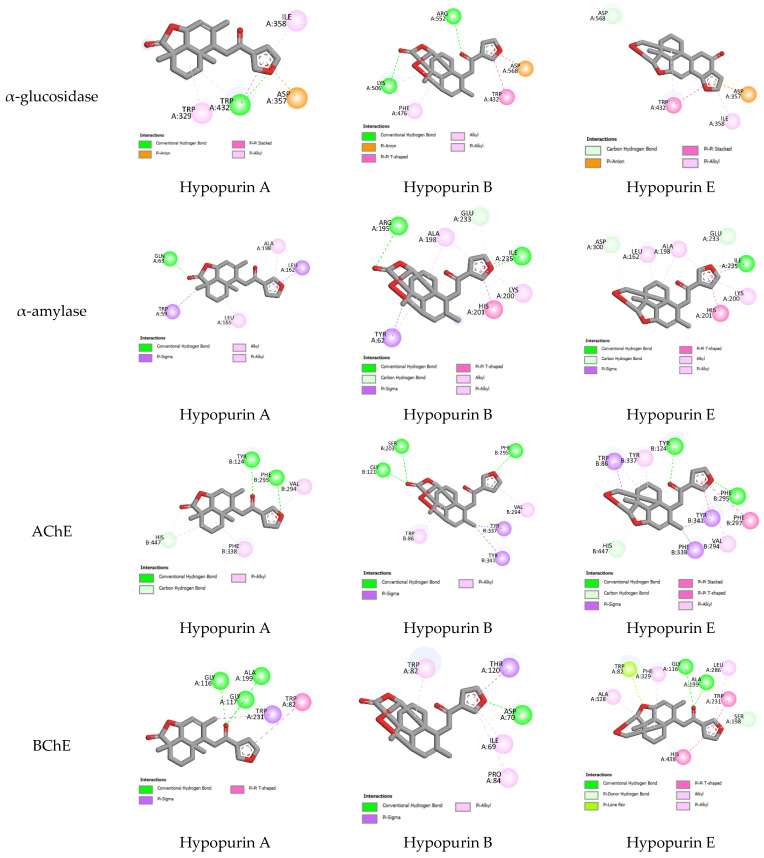
All 2D closest interactions between Hypopurin A, Hypopurin B and Hypopurin E compounds and active residues of α-amylase, α-glucosidase, acetylcholinesterase and butyrylcholinesterase.

The experimental anticholinesterase and antidiabetic assays of Hypopurin A, Hypopurin B and Hypopurin E are displayed in Table 1. All three compounds showed the ability to act as α-glucosidase, α-amylase, acetylcholinesterase and butyrylcholinesterase inhibitors. From the percentage inhibition measured and IC_50_ values, it appears that the inhibition of the isolated compounds may strongly depend on their structural features and their binding affinities into the binding sites of target enzymes (Table 1 and Figure 4). In an attempt to explain the observed inhibitions of Hypopurin A, Hypopurin B and Hypopurin E, molecular docking was performed to determine the binding modes between the tilted compounds from one side and the active residues of α-glucosidase, α-amylase, acetylcholinesterase and butyrylcholinesterase from the other side. Table 2 summarizes the free binding energies, the number of hydrogen bonds and the number of interactions in the complexes formed between the isolated compounds of Hypopurin A, Hypopurin B and Hypopurin E and the active residues of α-glucosidase, α-amylase, acetylcholinesterase and butyrylcholinesterase.

**Figure 5:**
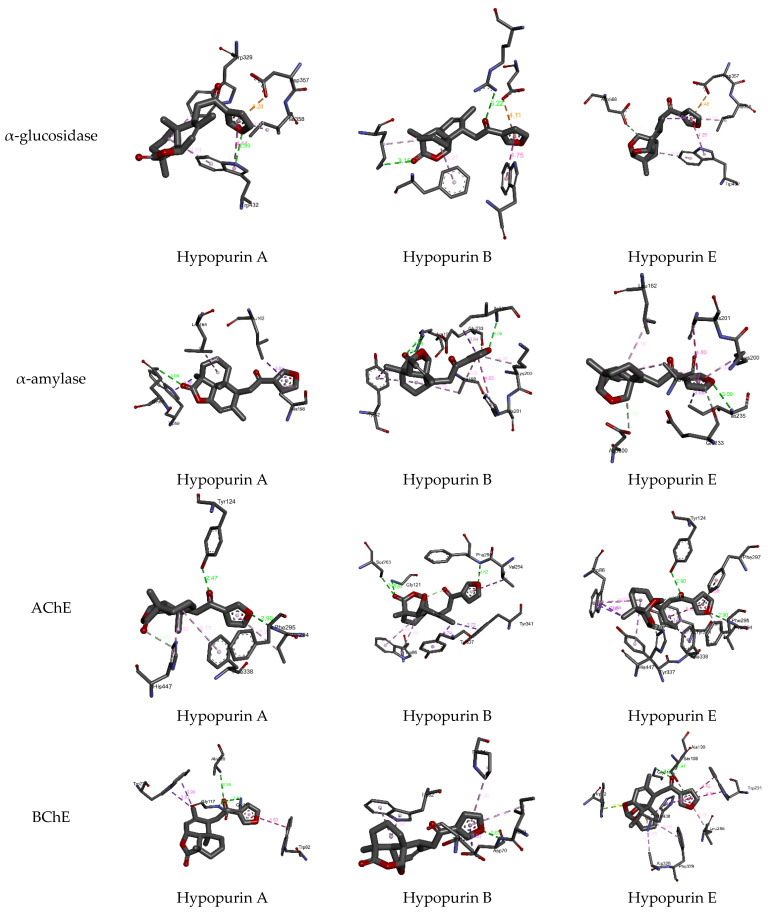
All 3D closest interactions between Hypopurin A, Hypopurin B and Hypopurin E compounds and active residues of α-amylase, α-glucosidase, acetylcholinesterase and butyrylcholinesterase. Characterization of isolated compounds.

From molecular docking outputs, Hypopurin A, Hypopurin B, and Hypopurin E compounds effectively fit into the binding sites of α-amylase, α-glucosidase, acetylcholinesterase and butyrylcholinesterase, forming stable complexes with negative bending energies (Table 1). The negative binding energies may indicate that α-glucosidase, α-amylase, acetylcholinesterase and butyrylcholinesterase inhibitions by Hypopurin A, Hypopurin B and Hypopurin E are thermodynamically favorable processes (Table 2). For each target, the binding energies of the stable complexes vary slightly, with maximal variations of 0.4, 0.52, 0.22 and 0.37 kcal mol^−1^ with respect to the stable complex formed into the binding sites of α-glucosidase, α-amylase, acetylcholinesterase and butyrylcholinesterase, respectively. Hence, the binding energy may not be considered a strong variable in distinguishing between the inhibition efficiencies of the tilted compounds. Therefore, our focus will be on the binding modes that they formed with the active residues of the target enzymes. Figure 4 and Figure 5 display 2D and 3D binding interactions of Hypopurin A, Hypopurin B and Hypopurin E into the binding sites of α-glucosidase, α-amylase, acetylcholinesterase and butyrylcholinesterase. 

## 3. Discussion

Acetylcholinesterase (AChE) and butyrylcholinesterase (BChE) catalyze the breaking down of acetylcholine, thereby lowering the levels of acetylcholine for neurotransmission and gradually decreasing cognitive functions. By inhibiting these two enzymes, the cholinergic transmission in the brain can be enhanced, thereby relieving the symptoms of AD, such as memory loss and mortality risk, making cholinesterase inhibitors the sole approved treatment for AD and neurodegenerative dementia [10,16,43]. Identifying effective long-term treatment for AD, as well as cholinesterases inhibitors with fewer side effects and high efficiency, is challenging, and natural products provide suitable alternatives to synthetic drugs [15]. The tested extract and isolated compounds exhibited moderate-togood inhibitions for both AChE and BChE, indicating their pharmacological potential in terms of the palliation of the symptoms and treatment of AD, as well as possibly reducing the progression of the disease by interfering with the cholinergic system. The isolated compounds, which are diterpenoids, triterpene and sterols, are described as classes of secondary metabolites with potential cholinesterase inhibition due to their small molecular sizes, and can cross the blood–brain barrier and exercise physiological effects [15,44,45,46]. Though AD cannot be cured easily, its progression can be slowed down or stopped. Multiple strategies, such as use of natural products, synthetic compounds and others options, are required to combat this chronic disease and its devastating effects on neurons and the cerebral cortex and hippocampus areas of the brain, which accounts for the cognitive impairment [47,48,49]. The evaluation for anticholinesterase activity of *G. pictum* can greatly contribute to AD drug development because the furanolabdanes show good potential to this effect. 

*G. pictum* is an important plant with various classes of phyto-constituents and bioactivities that is used for various medicinal purposes. In this study, important bioactive constituents are isolated and characterized, and surprisingly, furanolabdane diterpenoids are described for the first time in this plant. The isolation of furanolabdane diterpenoids from this plant for the first time is of great chemotaxonomic significance. The compounds and extractd showed potential to combat Alzheimer’s disease and diabetes, as they inhibited cholinesterases and carbohydrate digestive enzymes. Carbohydrates are digested into sugars by α-amylase and α-glucosidase, and this process leads to an increase in post-prandial glucose levels in the blood of diabetic patients, and substances which inhibit both enzymes are antidiabetic drug candidates [50,51]. Over 1200 plants and other natural products provide suitable medicines for the control and treatment of diabetes and obesity, and the search and development of new antidiabetic drugs from natural sources has great interest in terms of in vivo and in silico evaluations [52,53]. The antidiabetic potential exhibited by *G. pictum* and its compounds, especially the furanolabdanes, suggests that these constituents are responsible for the antidiabetic effects of the plant. The results are in conformity with other reports of antidiabetic effects of this plant, though these reports used different model experiments. *G. pictum* administered via the oral route effectively reduced blood glucose levels in alloxan-induced diabetes in mice at doses between 50 mg/kg and 200 mg/kg [54]. *G. pictum* extracts administered orally at 50 mg/kg over a period of 28 days were shown to decrease the glucose levels in blood by percentages ranging from 30 to 37% [55].

Enzymes intervene in metabolic reactions in the body, and imbalances in enzyme activity cause illnesses that can be corrected by blocking the enzyme activity [56,57]. Enzyme inhibition is, therefore, the basis of many drugs. The results for the inhibition of various enzymes indicate the effects of *G. pictum* on metabolic diseases, such as diabetes, and neurodegenerative diseases, such as AD, which are usually accompanied by oxidative stress [5,58]. In enzyme inhibitory assays, notably diabetic enzymes, the presence of a five-membered furan moiety in the furanolabdane diterpenoids Hypopurin E, Hypopurin B and Hypopurin A contributes to the inhibitory activity of the enzymes [59,60]. In some in silico evaluationa of α-glucosidase and AChE inhibitory activities, furanolabdane-type diterpenoids were evaluated through protein–ligand docking and molecular dynamics studies, which exhibited strong binding affinities towards the active site residues of the human-AChE enzyme, as well as low potency against the α-glucosidase enzyme [61].

For butyrylcholinesterase inhibition, Hypopurin A show higher inhibition efficiency than Hypopurin B and Hypopurin E. The higher inhibition efficiency of the former type may be linked to the number of hydrogen bonds that it forms with the active residues of butyrylcholinesterase (Figure 2 and Figure 3). For butyrylcholinesterase, binding affinity into the binding site of acetylcholinesterase reveals that Hypopurin E may have higher inhibition efficiency than Hypopurin A and Hypopurin B due to the great number of binding interactions formed by the former type compared to the latter types. Experimentally, Hypopurin A, Hypopurin B and Hypopurin E showed weak percentage inhibition efficiencies of less than 40%. For α-amylase, Hypopurin A shows higher inhibition efficiency compared to Hypopurin B and Hypopurin E, whereas molecular docking reveals that Hypopurin B may have potent inhibition efficiency (Figure 2 and Figure 3). For α-glucosidase, the higher inhibition efficiency of Hypopurin B follows molecular docking outputs (Figure 2 and Figure 3). Indeed, for α-glucosidase, Hypopurin B forms two strong hydrogen bonds with LYS A506 and ARG A552 of 3.15 and 3.22 Å, respectively. However, for Hypopurin B, only one hydrogen bond is formed with TRP A430 of 2.89 Å, while no hydrogen bond is formed with Hypopurin E (Figure 4 and Figure 5).

## 4. Materials and Methods

### 4.1. Instrumentation

Solvent evaporation was carried out using a Buchi Rotavapor (R215, Buchi, Flawil, Switzerland). Column chromatography (CC) was performed via glass column using silica gel (70-230 mesh, Merck, Darmstadt, Germany), and thin layer chromatography (TLC) was performed using silica gel pre-coated plates F-254 Merck (20 × 20 cm). Compounds were visualized under UV light (254 and 365 nm), sprayed with dilute sulfuric acid and heated. The melting points of the compounds were recorded using Buchi M-560 (Buchi, Flawil, Switzerland) melting point apparatus equipped with a Buchi M-569 sample loader. The ^1^H NMR (500 MHz) and ^13^C NMR (125 MHz) data were recorded via a Bruker Avance AV-500 (Bruker, Ettlingen, Germany) spectrometer in deuterated solvents, with trimethylsilane (TMS) used as the reference. Chemical shifts were given in ppm (δ), and coupling constants (J) in Hz. ESI-TOF-MS spectra were registered on a QTOF Spectrometer (Bruker, Ettlingen, Germany). A Multiskan Go microplate reader (Thermo Fischer Scientific, Waltham, MA, USA) was used to measure absorbances in the bioassays.

### 4.2. Chemicals

Acetylthiocholine iodide, butyrylthiocholine chloride, galantamine, 5,5′-dithiobis(2-nitrobenzoic) acid (DTNB), sodium carbonate (Na_2_CO_3_), butyrylcholinesterase (horse serum source) (EC 3.1.1.8) and acetylcholinesterase (electric eel source, type-VI-S, EC 3.1.1.7) were purchased from Sigma Chemical Co. (Sigma-Aldrich GmbH, Steinheim, Germany). The extraction solvents glutathione, Lugol, *p*-nitrophenyl-β-d-glucopyranoside (pNPG), α-amylase (from porcine pancreas), α-glucosidase (from Saccharomyces cerevisiae) and acarbose were obtained from Sigma-Aldrich Chemical Company (St. Louis, MO, USA). Silica gel (70–230 mesh) was purchased from Merck KGaA, Darmstadt, Germany.

### 4.3. Plant Material, Extraction and Isolation

The leaves and the stems of *Graptophyllum pictum* were harvested from Bameka locality, Hauts-Plateaux subdivision, West Region, Cameroon, in July 2018. A prepared voucher specimen of the plant was identified by the botanist Mr. Nana and deposited at the National Herbarium of Cameroon under the authentic voucher N° 66900HNC.

The leaves and the stems of *Graptophyllum pictum* were cut, before being dried in the absence of light for 3 weeks. They were then powdered. Next, 1 kg of each of the resulting powders were each extracted with 10 L of MeOH via maceration at room temperature. After filtration and concentration using a Rotary evaporator, 115 g and 110 g of crude extract were obtained from the leaves and stems, respectively. In total, 45 g of the crude leaves extract were purified on a silica gel column using an eluent gradient system n-hexane/AcOEt (100:0 to 0:100), followed by AcOEt/CH_3_OH (100:0 to 0:100); two pooled fractions A and B were then obtained after we regrouped the sub-fractions based on their TLC profiles. Fraction A (20.0 g) was eluted using a silica gel column on a gradient eluent system hexane/AcOEt (100:0 to 50:50) and afforded three furanolabdane diterpenoids: compound **3** (340 mg), compound **1** (97 mg) and compound **2** (94 mg). Fraction B (11.4 g) was purified using a silica gel column with n-hexane/AcOEt (10: 90) isocratic eluent, yielding compound **5** (110 mg). Next, 45 g of the crude stem extract was purified via column chromatography using silica gel column with an eluent gradient system n-hexane/AcOEt (100:0 to 0:100), followed by AcOEt/CH_3_OH (100:0 to 0:100), and six compounds were obtained as follows: compound **4** (34 mg), a mixture of compound **7a** and **7b** (53 mg), compound **1** (200 mg), compound **2** (3.9 mg), compound **6** (24.5 mg) and compound **5** (10 mg). 

### 4.4. Physical, Spectrometric and Spectroscopic Data of the Isolated Compounds 

The isolated compounds were identified from their physical, spectrometric and spectroscopic data, as summarized below. 

Hypopurin E (Compound **3**), White solid. Mp 119–120 °C. ^1^H NMR (Acetone-*d*_6_, 500 MHz): *δ*_H_ ppm 8.54 (1H, d, *J =* 1.2 Hz, H-16), 7.70 (1H, d*, J =* 18 Hz, H-15), 6.85 (1H, m, H-14), 5.76 (1H, d, *J =* 2.5 Hz, H-7), 4.79 (1H, s, H-20α), 4.37 (1H, t, *J* = 4.6 Hz, H-6), 3.74(1H, d, *J =* 2.7 Hz, H-20α), 3.38 (1H, d, *J =* 10.9 Hz, H-20β), 2.99 (1H, dd, *J* = 17, 8.5 Hz, H-11α), 2.86 (1H, d, *J =* 2.5 Hz, H-9β), 2.85 (1H, dd, *J =* 17*,* 2.5 Hz, H-11β) 2.05 (1H, dd, *J* = 2.5 Hz, H-2α), 1.88 (1H, m, H-1α), 1.80 (1H, m, H-3β), 1.63 (1H, s, H-5), 1.61 (1H, m, H-2β), 1.52 (1H, m, H-3α), 1.61 (3H, s, H-17), 1.20 (1H, m, H-1β), 0.96 (3H, s, H-19); ^13^C NMR (Acetone-*d*_6_, 125 MHz): *δ*_C_ 194.4 (C-12), 149.1 (C-16), 145.5 (C-15), 141.6 (C-8), 128.4 (C-13), 121.9 (C-7), 109.3 (C-14), 106.0 (C-18), 72.0 (C-6), 65.4 (C-20), 49.7 (C-5), 42.8 (C-9), 42.5 (C-4), 37.5 (C-11), 36.4 (C-10), 34.7(C-1), 31.7 (C-3), 23.7 (C-19), 22.1 (C-17), 21.2 (C-2); ESI-TOF-MS [M + H]^+^ *m*/*z* 329.7 for C_20_H_24_O_4._ HR-ESI-TOF-MS [M + Na]^+^ *m*/*z* 351.1572 g/mol (calculated for C_20_H_24_O_4_ + Na, *m*/*z* 351.1572).

Hypopurin B (Compound **2**), White solid. Mp 120–121.5 °C. ^1^H NMR (Acetone-*d*_6_, 500 MHz): *δ*_H_ ppm 8.61 (1H, s, H-16), 7.72 (1H, s*,* H-15), 6.88 (1H, d, *J =* 0.4 Hz, H-14), 5.90 (1H, d, *J =* 6.0 Hz, H-7), 5.54 (1H, s, H-20), 4.59 (1H, d, *J* = 6.0 Hz, H-6), 3.21 (1H, d, *J =* 8.0 Hz, H-9), 3.14 (1H, dd, *J =* 17, 8.5 Hz, H-11°), 2.81 (1H, dd, *J* = 17, 2.5 Hz, H-11b), 2.08 (1H, s, H-5),1.67 (1H, m, H-1*β*), 2.05 (1H, dd, *J* = 2.5 Hz, H-2α), 1.73 (1H, m, H-3*β*), 1.74 (1H, m, H-2β), 1.48 (1H, m, H-3α), 1.46 (3H, s, H-17), 1.29 (3H, s, H-19); ^13^C NMR (Acetone-*d*_6_, 125 MHz): *δ_C_* 193.7 (C-12), 176.0 (C-18),149.7 (C-16), 145.7 (C-15), 142.0 (C-8), 128.5 (C-13), 127.5 (C-7), 109.3 (C-14), 106.1 (C-20), 74.2 (C-6), 54.5 (C-5), 47.6 (C-10), 46.9 (C-9), 44.8 (C-4), 40.0 (C-11), 28.8 (C-3), 38.9 (C-1), 22.1 (C-19), 20.7 (C-17), 21.3 (C-2); ESI-TOF-MS *m*/*z* 365.6 [M + Na]^+^ for C_20_H_22_O_5_.

Hypopurin A (Compound **1**), Gray solid. mp 125–126 °C. ^1^H NMR (Acetone-*d*_6_, 500 MHz): *δ*_H_ ppm 8.07 (1H, s, H-16), 7.45 (1H, s, H-15), 6.78 (1H, d, *J* = 2.0 Hz, H-14), 5.89 (1H, d, *J =* 4.0 Hz, H-7), 4.85 (1H, s, H-6), 2.93 (1H, m, H-9), 2.91 (1H, m, H-11α), 2.60 (1H, dd, *J* = 22, 7.5 Hz, H-11β), 2.10 (1H, dd, *J =* 14, 4.5 Hz, H-3*α*), 1.87 (1H, d, *J* = 5.0 Hz, H-5), 1.70 (1H, m, H-2*α*), 1.62 (3H, s, H-17), 1.53 (1H, m, H-2α), 1.51–1.42 (2H, m, H-1*α* and H-3α), 1.30 (1H, m, H-1β), 1.31 (3H, s, H-19), 0.83 (3H, s, H-20). ^13^C NMR (Acetone-*d*_6_, 125 MHz): *δ_C_* 194.7 (C-12), 182.3 (C-18),149.1 (C-16), 145.5 (C-15), 144.7 (C-8), 128.5 (C-13), 120.6 (C-7), 109.3 (C-14), 73.9 (C-6), 51.3 (C-5), 46.2 (C-10), 43.4 (C-4), 38.0 (C-1), 34.1 (C-11), 29.0 (C-3), 24.2 (C-9), 19.0 (C-19), 18.7 (C-17), 18.7 (C-2); 14.3 (C-20).

Lupeol (Compound **4**), White solid. m.p. 212–213 °C. ^1^H NMR (CDCl_3_, 500 MHz): *δ*_H_ ppm 0.79 (s, Me-25), 0.82 (s, Me-24), 0.86 (s, Me-26), 0.97 (s, Me-27), 1.00 (s, Me-23), 1.71 (s, Me-30), 2.40 (1H, td, *J* = 11.1 and 5.8 Hz, H-19), 3.21 (1H, dd, *J* = 11.4 and 4.9 Hz, H-3), 4.60 (1H, d, *J* = 2.4Hz, H-29a), 4.72 (1H, d, *J* = 2.4 Hz, H-29b); ^13^C NMR (CDCl_3_, 125 MHz): *δ*_C_ 38.5 (C-1), 27.5 (C-2), 79.0 (C-3), 38.7 (C-4), 55.3 (C-5), 18.2 (C-6), 33.2 (C-7), 41.9 (C-8), 50.2 (C-9), 37.4 (C-10), 20.6 (C-11), 23.7 (C-12), 32.5 (C-13), 42.5 (C-14), 27.5 (C-15), 40.4 (C-16), 48.6 (C-17), 53.8 (C-18), 48.0 (C-19), 151.0 (C-20), 27.6 (C-21), 44.5(C-22), 28.2 (C-23), 15.7 (C-24), 16.8 (C-25), 16.1 (C-26), 15.3 (C-27), 16.8 (C-28), 109.3 (C-29), 19.4 (C-30). 

3*β*-*O*-D-glucopyranosyl-*β*-sitosterol (Compound **5**), White powder. m.p. 175–177 °C. ^1^H NMR (CDCl_3_, 500 MHz): *δ*_H_ ppm 0.74 (s, Me-19), 0.81 (d, *J* = 6.7 Hz, Me-26), 0.83 (d, *J* = 6.7 Hz, Me-27), 0.86 (t, *J* = 7.2 Hz, Me-28), 0.92 (d, *J* = 6.5 Hz, Me-21), 1.05 (s, Me-18); 3.53 (1H, tdd, *J* = 4.6, 4.5 and 3.7 Hz, H-3), 4.98 (1H, m, H-22), 5.14 (1H, m, H-23), 5.34 (1H, t, *J* = 6.5 Hz, H-5); ^13^C NMR (CDCl_3_, 125 MHz): *δ*_C_ 36.8 (C-1), 40.0 (C-2), 72.0 (C-3), 42.3 (C-4), 140.8 (C-5), 120.9 (C-6), 30.7 (C-7), 31.9 (C-8), 50.2 (C-9), 35.5 (C-10), 21.1 (C-11), 39.7 (C-12), 42.2 (C-13), 56.9 (C-14), 24.4 (C-15), 28.9 (C-16), 56.0 (C-17), 19.4 (C-18), 12.1 (C-19), 39.5 (C-20), 21.1 (C-21), 32.7 (C-22), 24.8 (C-23), 51.3 (C-24), 31.9 (C-25), 21.2 (C-26), 19.0 (C-27), 25.4 (C-28), 12.3 (C-29). 102.3 (C-1′), 75.4 (C-2′), 78.7 (C-3′), 71.8 (C-4′), 78.6 (C-5′), 62.9 (C-6′).

Stigmasterol 3-*O*-β-d-glucopyranoside (Compound **6**): White powder. m.p. 175–176 °C. ^1^H NMR (CDCl_3_, 500 MHz): *δ*_H_ ppm 0.74 (s, Me-19), 0.81 (d, *J* = 6.7 Hz, Me-26), 0.83 (d, *J* = 6.7 Hz, Me-27), 0.86 (t, *J* = 7.2 Hz, Me-28), 0.92 (d, *J* = 6.5 Hz, Me-21), 1.05 (s, Me-18); 3.53 (1H, tdd, *J* = 4.6, 4.5 and 3.7 Hz, H-3), 4.98 (1H, m, H-22), 5.14 (1H, m, H-23), 5.34 (1H, t, *J* = 6.5 Hz, H-5); ^13^C NMR (CDCl_3_, 125 MHz): *δ*_C_ 37.2 (C-1), 31.9 (C-2), 71.8 (C-3), 42.3 (C-4), 140.8 (C-5), 121.7 (C-6), 31.7 (C-7), 31.9 (C-8), 50.2 (C-9), 36.5 (C-10), 21.1 (C-11), 39.7 (C-12), 42.2 (C-13), 56.9 (C-14), 24.4 (C-15), 28.9 (C-16), 56.0 (C-17), 19.4 (C-18), 12.1 (C-19), 40.5 (C-20), 21.1 (C-21), 138.3 (C-22), 129.3 (C-23), 51.3 (C-24), 31.9 (C-25), 21.2 (C-26), 19.0 (C-27), 25.4 (C-28), 12.3 (C-29). 102.3 (C-1′), 75.4 (C-2′), 78.7 (C-3′), 71.8 (C-4′), 78.6 (C-5′), 62.9 (C-6′).

β-sitosterol (Compound **7a**), White powder. ^1^H NMR (CDCl_3_, 500 MHz): *δ*_H_ ppm 0.73 (s, Me-19), 0.80 (d, *J* = 6.7 Hz, Me-26), 0.81 (d, *J* = 6.7 Hz, Me-27), 0.87 (t, *J* = 7.2 Hz, Me-28), 0.92 (d, *J* = 6.5 Hz, Me-21), 1.05 (s, Me-18); 3.53 (1H, tdd, *J* = 4.6, 4.5 and 3.7 Hz, H-3), 4.98 (1H, m, H-22), 5.14 (1H, m, H-23), 5.34 (1H, t, *J* = 6.5 Hz, H-5); ^13^C NMR (CDCl_3_, 125 MHz): *δ*_C_ 36.4 (C-1), 31.8 (C-2), 71.8 (C-3), 42.2 (C-4), 140.8 (C-5), 121.8 (C-6), 32.9 (C-7), 30.9 (C-8), 50.1 (C-9), 36.5 (C-10), 21.1 (C-11), 39.7 (C-12), 42.3 (C-13), 56.9 (C-14), 24.4 (C-15), 28.9 (C-16), 56.1 (C-17), 19.4 (C-18), 19.1 (C-19), 36.2 (C-20), 18.1 (C-21), 34.0 (C-22), 26.1 (C-23), 51.3 (C-24), 29.2 (C-25), 19.7 (C-26), 19.0 (C-27), 25.4 (C-28), 12.3 (C-29).

Stigmasterol (Compound **7b**), White powder. ^1^H NMR (CDCl_3_, 500 MHz): *δ*_H_ ppm 0.73 (s, Me-19), 0.80 (d, *J* = 6.7 Hz, Me-26), 0.81 (d, *J* = 6.7 Hz, Me-27), 0.87 (t, *J* = 7.2 Hz, Me-28), 0.92 (d, *J* = 6.5 Hz, Me-21), 1.05 (s, Me-18); 3.53 (1H, tdd, *J* = 4.6, 4.5 and 3.7 Hz, H-3), 4.98 (1H, m, H-22), 5.14 (1H, m, H-23), 5.34 (1H, t, *J* = 6.5 Hz, H-5); ^13^C NMR (CDCl_3_, 125 MHz): *δ*_C_ 36.4 (C-1), 40.4 (C-2), 71.8 (C-3), 50.4 (C-4), 140.8 (C-5), 121.7 (C-6), 33.9 (C-7), 31.9 (C-8), 42.2 (C-9), 40.6 (C-10), 21.1 (C-11), 32.0 (C-12), 42.2 (C-13), 51.5 (C-14), 23.1 (C-15), 24.3 (C-16), 56.9 (C-17), 19.4 (C-18), 21.2 (C-19), 37.5 (C-20), 12.1 (C-21), 129.3 (C-22), 138.3 (C-23), 56.3 (C-24), 33.9 (C-25), 19.5.2 (C-26), 19.0 (C-27), 25.4 (C-28), 12.3 (C-29).

### 4.5. Anticholinesterase Activity Assay

The anticholinesterase activity of the extracts and compounds was determined spectrophotometrically via the acetylcholinesterase (AChE) and butyrylcholinesterase (BChE) enzyme inhibition assay method described by Ellman with minor modifications [62,63]. AChE and BChE from electric eel and horse serum, respectively, were used. The substrates of the reactions were acetylthiocholine iodide and butyrylthiocholine chloride. The activity of the cholinesterase was monitored using DTNB (5,5′-Dithio-bis(2-nitrobenzoic) acid). Galantamine served as a standard compound. A Multiskan Go microplate reader spectrophotometer (Thermo Fischer Scientific, Waltham, MA, USA) was used to read optical densities.

### 4.6. Antidiabetic Activity Assay

The inhibition of α-amylase was determined using starch–iodine method with few modifications [64,65]. Next, 50 µL of α-amylase from porcine pancreas in pH 6.9 phosphate buffer prepared with 6 mM NaCl and 25 µL of sample solutions was mixed in a 96-well microplate. The mixture was pre-incubated for 10 min at 37 °C. Next, 50 µL of starch solution (0.05%) was added and incubated for 10 min at 37 °C. Following incubation, the reaction was completed by adding HCl (0.1 M, 25 µL) and Lugol (100 µL) solutions, and the absorbance was recorded at 630 nm using a Multiskan Go microplate reader spectrophotometer (Thermo Fischer Scientific, Waltham, MA, USA).

The α-glucosidase inhibitory activity was evaluated according to the method described previously [66]. A total of 50 µL of glutathione, 50 µL of sample solution, 50 µL of α-glucosidase from *Saccharomyces cerevisiae* in phosphate buffer (0.01 M pH 6.8) and 50 µL of PNPG (4-*N*-nitrophenyl-α-d-glucopyranoside) in phosphate buffer (0.01 M pH 6.8) were mixed in a 96-well microplate. The solution was incubated for 15 min at 37 °C. The reaction was then stopped with the addition 50 μL of sodium carbonate (0.2 M), and absorbances were read at 400 nm using a Multiskan Go microplate reader spectrophotometer (Thermo Fischer Scientific, Waltham, MA, USA). Acarbose was used as a standard compound for both analyses. Results were given as the percentage inhibition (%) at 100 µg/mL and 50% inhibition concentration (IC_50_).

### 4.7. Molecular Docking Details

The binding affinities of Hypopurin A, Hypopurin B and Hypopurin E to the binding sites of α-glucosidase, α-amylase, acetylcholinesterase, and butyrylcholinesterase were performed using the Autodock4 package [67]. X-ray coordinates of the targeted *α*-glucosidase, α-amylase, acetylcholinesterase and butyrylcholinesterase, as well as their corresponding original docked ligands, were downloaded from the RCSB data bank website with PDB codes 3W37, 1B2Y, 4EY7 and 1P0I, respectively [68,69,70,71]. Water molecules were removed, and polar hydrogen atoms and Kollman charges were added to the extracted receptor structure, i.e., α-glucosidase, α-amylase, acetylcholinesterase and butyrylcholinesterase, using the automated using AutoDock Tools 4.2. The active sites were identified based on co-crystallized receptor–ligand complex structures of α-glucosidase, α-amylase, acetylcholinesterase and butyrylcholinesterase. The re-docking of the original ligands into the binding active site of α-glucosidase, α-amylase, acetylcholinesterase and butyrylcholinesterase were well reproduced, achieving RMSD values of 0.99, 1.01, 0.63 and 0.82 Å, respectively. Molecular geometries of Hypopurin A, Hypopurin B, and Hypopurin E were minimized using Merck molecular force field 94 (MMFF94) level44 and saved as PDB files. The molecular docking study was performed using the Lamarckian genetic algorithm, with 500 being the total number of runs for binding sites. In each respective run, a population of 150 individuals with 27,000 generations and 250,000 energy evaluations was employed. Operator weights for crossover, mutation and elitism were set to 0.8, 0.02, and 1, respectively. The grid box centered at (0.067, −1.695, −22.993), (17.388, 5.268, 46.733), (10.634, −56.163, −23.873) and (138.693, 116.26, 40.971) with dimensions of 40 × 46 × 40, 44 × 40 × 40, 40 × 40 × 40 and 40 × 40 × 40 points, as well as spacing of 0.375 Å, were chosen for *α*-glucosidase, *α*-amylase, acetylcholinesterase and butyrylcholinesterase, respectively. The binding interactions between the docked Hypopurin A, Hypopurin B, and Hypopurin E into the binding sites of α-glucosidase, α-amylase, acetylcholinesterase and butyrylcholinesterase were visualized through the Discovery Studio Client (Discovery Studio Client is A Product of Accelrys Inc., San Diego, CA, USA).

### 4.8. Statistical Analyses

Each experiment was performed in triplicate. The results are expressed as mean ± standard error of the mean. Student’s test was used to determine the significant differences between various means, and values of *p* < 0.05 were regarded as significant.

## 5. Conclusions

The medicinal plant *G. pictum* was used to treat various ailments in many parts of the world, especially in the Ayurvedic traditional medicine systems in Asian countries. This plant contains bioactive compounds. In this study, aerial parts of *G. pictum* were subjected to column chromatography, and pure compounds were isolated, especially the furanolabdane diterpenoids Hypopurin A, Hypopurin B and Hypopurin E. Specific inhibitors of some enzymes were developed as medication for diseases such as hyperglycemia and Alzheimer’s disease. The extracts and pure compounds were evaluated for their inhibitory effects against glucose digestive enzymes α-amylase and α-glucosidase, exhibiting good activity. Equally, the compounds and extracts inhibited cholinesterases (AChE and BChE). Additionally, the effects of functional groups and structural features of the furanolabdanes on the enzyme inhibitory potential was evaluated, and structure–activity relationships were deduced. The molecular docking studies shed more light on the binding properties of the compounds and further explained the observed activities. In all enzyme inhibitions, the extracts and the furanolabdanes were active, and the results indicated that *G. pictum* and its phyto-consituents could be used in the development of Alzheimer’s disease therapies and antidiabetic drugs. It will be of interest to further investigate the molecular effects, toxicity and mechanisms of action of the potent compounds in future works. 

## Figures and Tables

**Table 1:** IC_50_ values (µg/mL) of samples in anticholinesterase and antidiabetic assays.

Test Sample	Anticholinesterase Activity		Antidiabetic Activity	
	AChE	BChE	α-Glucosidase	α-Amylase
**Leaf extract**	>100	58.21 ± 0.65	48.90 ± 0.17	70.21 ± 0.53
**Stem extract**	>100	67.05 ± 0.82	45.61 ± 0.56	64.47 ± 0.78
**Hypopurin A**	>100	58.00 ± 0.90	99.25 ± 0.96	60.68 ± 0.55
**Hypopurin B**	>100	67.05 ± 0.92	71.41 ± 0.98	69.51 ± 1.30
**Hypopurin E**	>100	86.90 ± 0.76	76.33 ± 1.10	73.80 ± 0.75
**Lupeol**	>100	>100	69.75 ± 0.42	>100
**β-Sitosterol glucoside**	>100	>100	>100	>100
**Stigmasterol glucoside**	>100	>100	>100	>100
**Stigmasterol and β-sitosterol**	>100	>100	>100	>100
**Galantamine**	5.50 ± 0.25	42.27 ± 0.22	NT	NT
**Acarbose**	NT	NT	87.70 ± 0.68	32.25 ± 0.36

Values represent mean ± SEM of three parallel sample measurements (*p* < 0.05). NT: not tested.

**Table 2:** Free binding energies, hydrogen bonding and number of closest residues to docked Hypopurin A, Hypopurin B and Hypopurin E into binding sites of α-glucosidase, α-amylase, AChE and BChE and IC_50_ values.

Compound	Free Binding Energy (kcal/mol)	H-Bonds (HBs)	Number of Closest Residues to the Docked Ligand in the Active Site	IC_50_ ± SEM
α-glucosidase				
Hypopurin A	−7.61	1	6	99.25 ± 0.96
Hypopurin B	−7.21	2	6	71.41 ± 0.98
Hypopurin E	−7.25	0	5	76.33 ± 1.10
α-amylase				
Hypopurin A	−8.03	1	5	60.68 ± 0.55
Hypopurin B	−8.33	2	10	69.51 ± 1.30
Hypopurin E	−7.81	1	9	73.80 ± 0.75
Acetylcholinesterase				
Hypopurin A	−10.17	2	5	>100
Hypopurin B	−10.29	3	7	>100
Hypopurin E	−10.39	2	11	>100
Butyrylcholinesterase				
Hypopurin A	−8.77	2	4	58.00 ± 0.90
Hypopurin B	−8.43	1	5	67.05 ± 0.92
Hypopurin E	−8.80	2	10	86.90 ± 0.76

## Data Availability

The data supporting reported results can be obtained from the corresponding author upon reasonable request.

## Supplementary Materials

The following supporting information can be downloaded at: https://www.mdpi.com/article/10.3390/molecules28124802/s1, the spectral data of compound **3** (Hypopurin E) are provided as supporting information in Figure S1: elemental composition report; Figure S2: HR-ESI-TOF-MS; Figure S3: ^1^H NMR spectrum (600 MHz, acetone-*d*_6_); Figure S4: ^1^H NMR spectrum (600 MHz, acetone-*d_6_*) of compound **3** (enlarged 0.5–4.0 ppm); Figure S5: DEPT spectrum; Figure S6: ^13^C NMR spectrum (150 MHz, acetone-*d*_6_); Figure S7: HSQC spectrum; Figure S8: HMBC spectrum; Figure S9: ^1^H-^1^H COSY spectrum; Figures S10–S13: graphs of standards for bioassays; Tables S1 and S2: equations for determination of IC_50_ for test samples.

## Sample Availability

Samples of the compound **3** (Hypopurin E) are available from the authors on request.
