# Supplementary material for: In Vitro and In Silico Evaluation of Anticholinesterase and Antidiabetic Effects of Furanolabdanes and Other Constituents from Graptophyllum pictum (Linn.) Griffith
Source: Molecules. 2023 Jun 16;28(12):4802. doi: 10.3390/molecules28124802 (PMC10304387; doi:10.3390/molecules28124802)
Supplement: Supplementary file 1 [file molecules-28-04802-s001.zip › molecules-2402975-supplementary.pdf]

In vitro and *in silico* evaluation of anticholinesterase and antidiabetic effects of furanolabdanes and other constituents from *Graptophyllum pictum* (Linn.) Griffith

Nathalie Tanko Metiefeng[1], Alfred Ngenge Tamfu[2,3,4], Maurice Fotsing Tagatsing[1], Turibio Kuiate Tabopda[1], Selcuk Kucukaydin[3], Martin Noah Mbane[1], Alex de Theodore Atchade[1], Emmanuel Talla[2], Celine Henoumont[5], Sophie Laurent[5], El Hassane Anouar[6], Rodica Mihaela Dinica[4]

[1]*Department of Organic Chemistry, Faculty of Science, The University of Yaounde I, Yaounde, 812, Cameroon*
[2]*Department of Chemical Engineering, School of Chemical Engineering and Mineral Industries, University of Ngaoundere, 454 Ngaoundere, Cameroon*
[3]*Department of Medical Services and Techniques, Koycegiz Vocational School of Health Services, Mugla Sitki Kocman University, 48800 Mugla, Turkey*
[4]*Department of Chemistry, Physics and Environment, Faculty of Sciences and Environment, 'Dunarea de Jos University', Galati, 47 Domneasca Str., 800008, Galati, Romania*
[5]*Laboratory of NMR and Molecular Imaging, Department of General, Organic Chemistry and Bio-medical, University of Mons, B- 7000, Mons, Belgium*
[6]*Department of Chemistry, College of Sciences and Humanities in Al-Kharj, Prince Sattam bin Abdulaziz University, Al-Kharj, Saudi Arabia*
Correspondence: macntamfu@yahoo.co.uk (A.N. Tamfu) ; rodica.dinica@ugal.ro (R.M. Dinica)

Abstract: *Graptophyllum pictum* is a tropical plant noticeable for its variegated leaves and usually exploited for various medicinal purposes. In this study, seven compounds including three furanolabdane diterpenoids which were Hypopurin E, described for the first time, Hypopurin A and Hypopurin B together with Lupeol, β-sitosterol 3-O-β-D-glucopyranoside, stigmasterol 3-O-β-D-glucopyranoside and a mixture of β-sitosterol and stigmasterol were isolated from *G. pictum* and their structures deduced from ESI-TOF-MS, HR-ESI-TOF-MS, 1D and 2D NMR experiments. The compounds were evaluated for their anticholinesterase activities against acetylcholinesterase (AChE) and butyrylcholinesterase (BChE) as well as their antidiabetic potential through inhibition of α-glucosidase and α-amylase. For AChE inhibition, no sample had IC50 within tested concentrations but the most potent was Hypopurin A with percentage inhibition of 40.18±0.75% compared to galantamine (85.91±0.58%) at 100 μg/mL. BChE was more susceptible to the leaves extract (IC_{50} = 58.21±0.65 μg/mL), stem extract (IC_{50} = 67.05±0.82 μg/mL), Hypopurin A (IC_{50} = 58.00±0.90 μg/mL), Hypopurin B (IC_{50} = 67.05±0.92 μg/mL) and Hypopurin E (IC_{50} = 86.90±0.76 μg/mL). In the antidiabetic assay, the furanolabdane diterpenoids, lupeol and the extracts had moderate to good activities. Against α-glucosidase, lupeol, Hypopurin E, Hypopurin A and Hypopurin B had appreciable activities but the leaves (IC_{50} = 48.90±0.17 μg/mL) and stem (IC_{50} = 45.61±0.56 μg/mL) extracts were more active than the pure compounds. In the α-amylase assay, stem extract (IC_{50} = 64.47±0.78 μg/mL), Hypopurin A (IC_{50} = 60.68±0.55 μg/mL) and Hypopurin B (IC_{50} = 69.51±1.30 μg/mL) had moderate activities compared to the standard acarbose (IC_{50} = 32.25±0.36 μg/mL). Molecular docking was been performed to determine the binding modes and free binding energies of Hypopurin E, Hypopurin A and Hypopurin B to the enzymes and decipher structure-activity relationship. The results indicated that G. pictum and its compounds could more or less be used in the development of therapies for Alzheimer's disease and diabetes.

Keywords: *Graptophyllum pictum*; furanolabdanes; anticholinesterase; α-amylase; α-glucosidase; molecular docking

List of figures in supplementary data

Figure S1: Elemental composition report of Compound 3

Figure S2: HR-ESI-TOF-MS of Compound 3

Figure S3: ^{1}H NMR spectrum (600 MHz, acetone-d_6) of compound 3

Figure S4: ^{1}H NMR spectrum (600 MHz, acetone-d_6) of compound 3 (enlarged 0.5 - 4.0 ppm)

Figure S5: DEPT spectrum of compound 3

Figure S6: ^{13}C NMR spectrum (150 MHz, acetone-d_6) of compound 3

Figure S7: HSQC spectrum of compound 3

Figure S8: HMBC spectrum of compound 3

Figure S9: ^{1}H-^{1}H COSY spectrum of compound 3

Figure S10: Galantamine graph for IC_{50} calculation of the acetylcholinesterase (AChE) enzyme inhibition (Concentration range 3.125 – 12.5 μg/mL)

Figure S11: Galantamine graph for IC_{50} calculation of the acetylcholinesterase (AChE) enzyme inhibition (Concentration range 12.5 – 100 μg/mL)

Figure S12: Acarbose graph for IC_{50} calculation of the α-glucosidase enzyme inhibition (Concentration range 12.5 – 100 μg/mL)

Figure S13: Acarbose graph for IC_{50} calculation of the α-glucosidase enzyme inhibition (Concentration range 25 – 100 μg/mL)

Table S1: Equations used for IC_{50} calculation of the anticholinesterase activity of the samples (Concentration range 25 – 100 μg/mL)

Table S2: Equations used for IC_{50} calculation of the antidiabetic activity of the samples (Concentration range 25 – 100 μg/mL)

Elemental Composition Report

Single Mass Analysis

Tolerance = 20.0 PPM / DBE: min = -1.5, max = 50.0
Element prediction: Off
Number of isotope peaks used for i-FIT = 3

Monoisotopic Mass, Even Electron Ions
7 formula(e) evaluated with 1 results within limits (all results (up to 1000) for each mass)
Elements Used:
C: 15-25 H: 15-30 O: 2-6 Na: 1-1

SYNAPT_PHIL_230421_RMN_GF03_01 129 (2.198) AM (Cen,2, 80.00, Ar,10000.0,472.67,0.00); Sm (SG, 3x2.00); Cm (1:143-89:138)

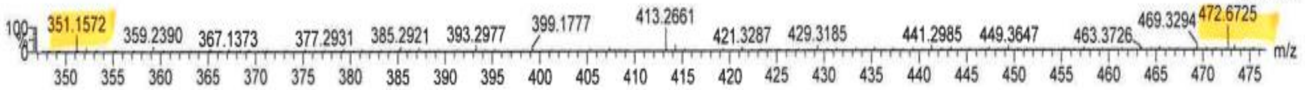

Mass	Calc. Mass	mDa	PPM	DBE	i-FIT	Norm	Conf(%)	Formula
Minimum:				-1.5				
Maximum:		5.0	20.0	50.0				
351.1572	351.1572	0.0	0.0	8.5	776.7	n/a	n/a	C20 H24 O4 Na

Figure S1: Elemental composition report of Compound 3

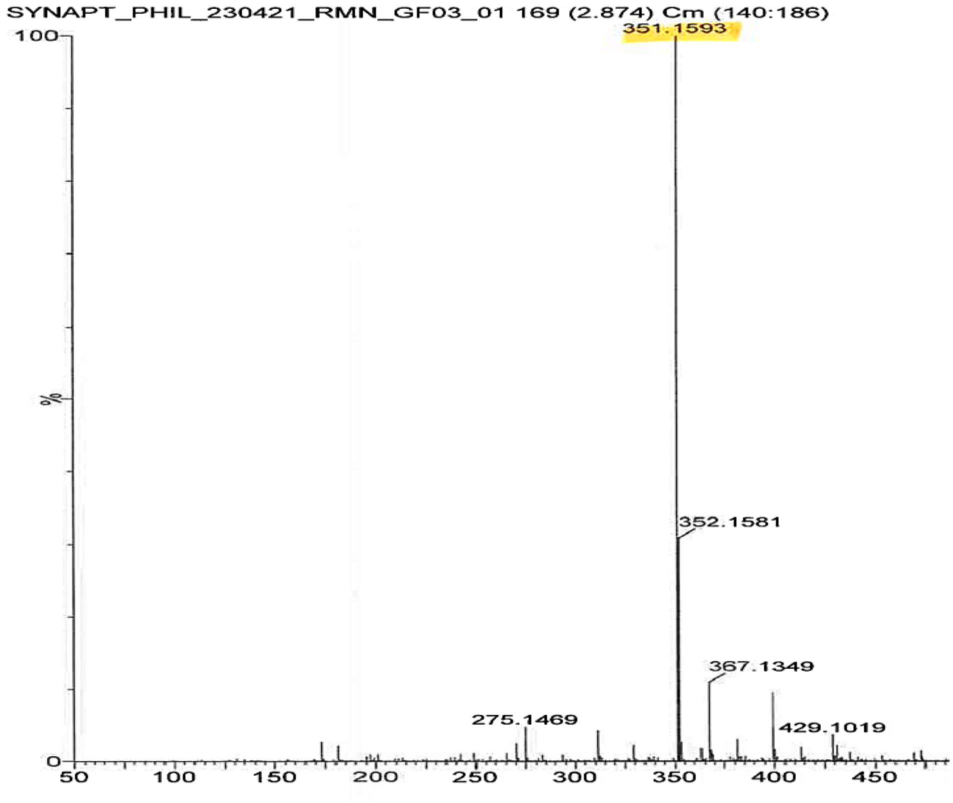

Figure S2: HR-ESI-TOF-MS of Compound 3

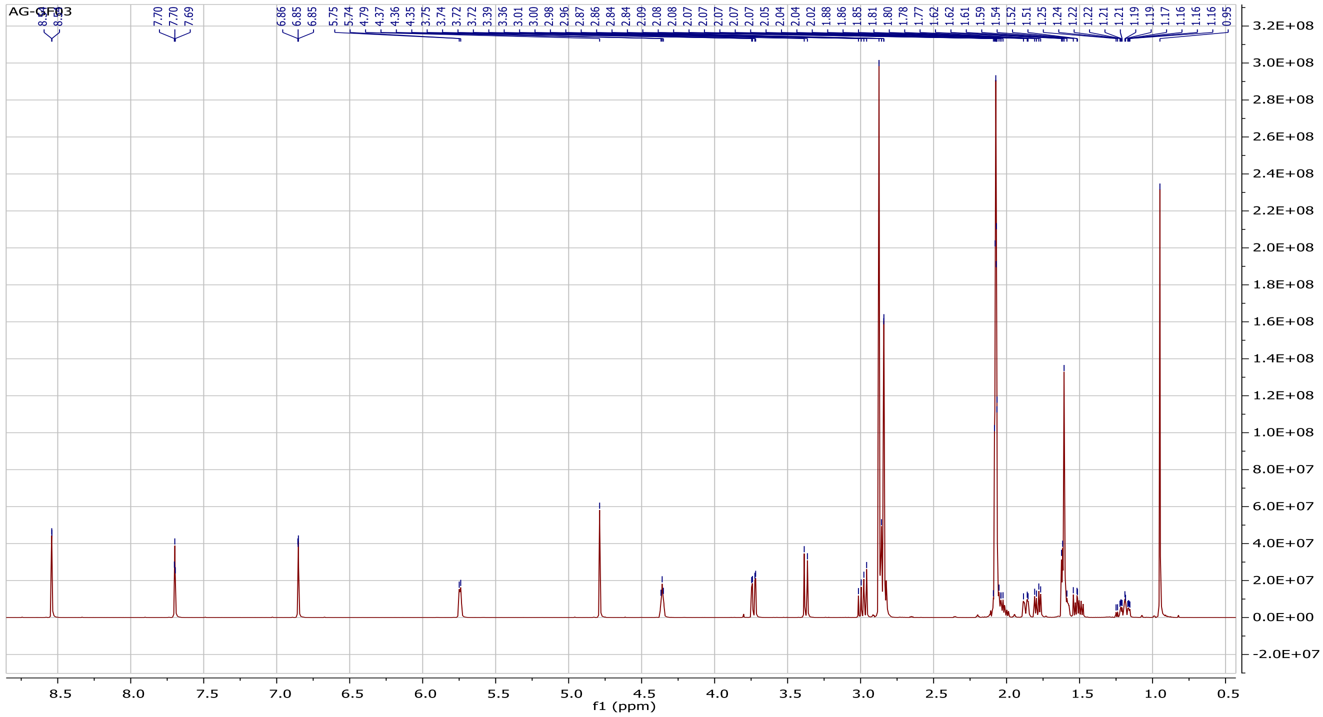

Figure S3: ^{1}H NMR spectrum (600 MHz, acetone-*d₆*) of compound 3

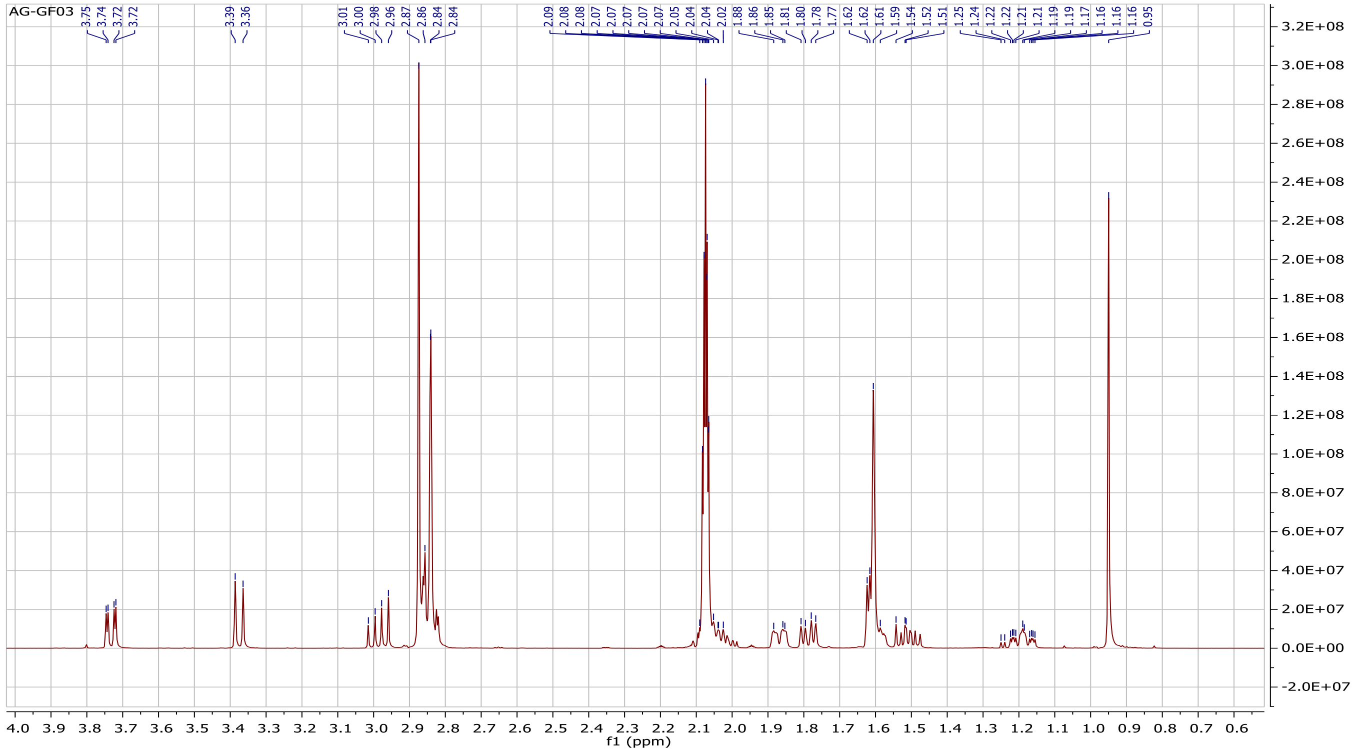

Figure S4: ^{1}H NMR spectrum (600 MHz, acetone-*d₆*) of compound 3 (enlarged 0.5 - 4.0 ppm)

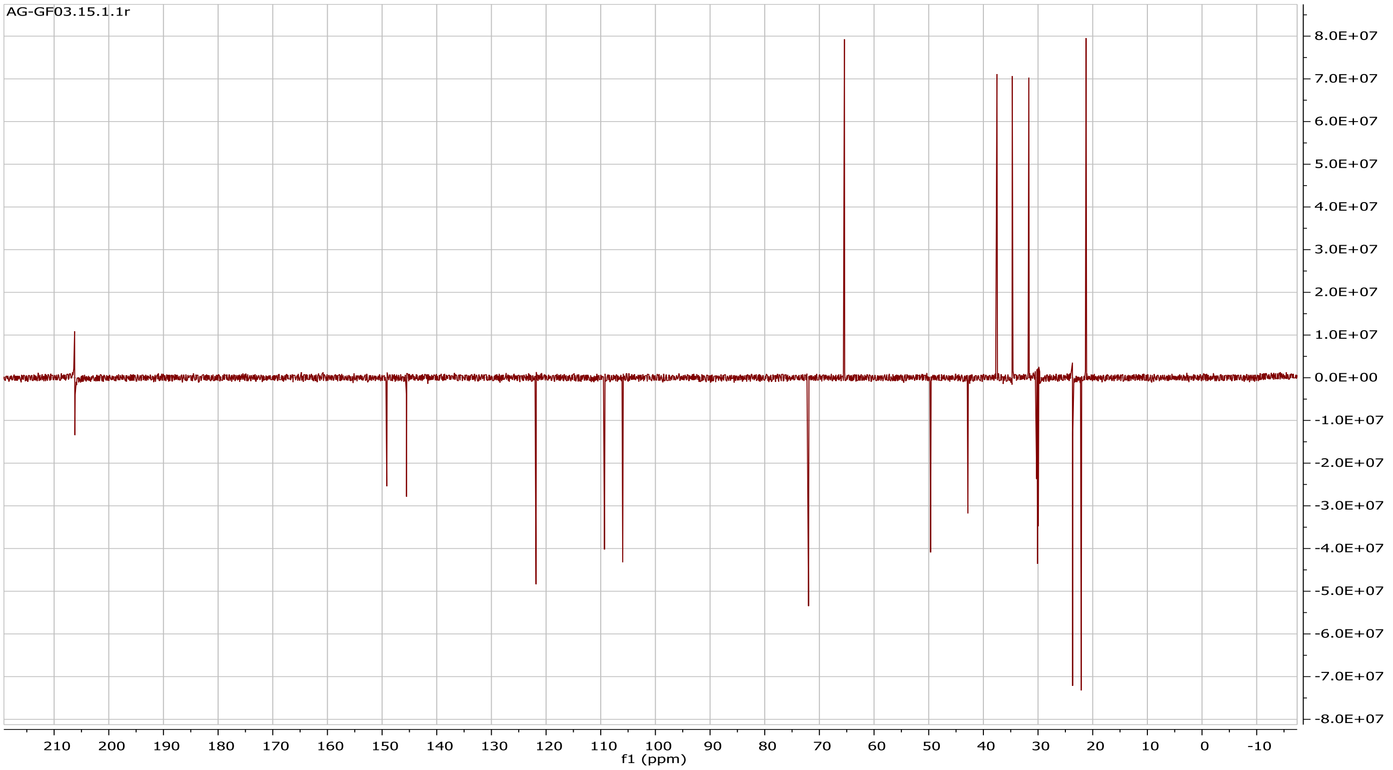

Figure S5: DEPT spectrum of compound 3

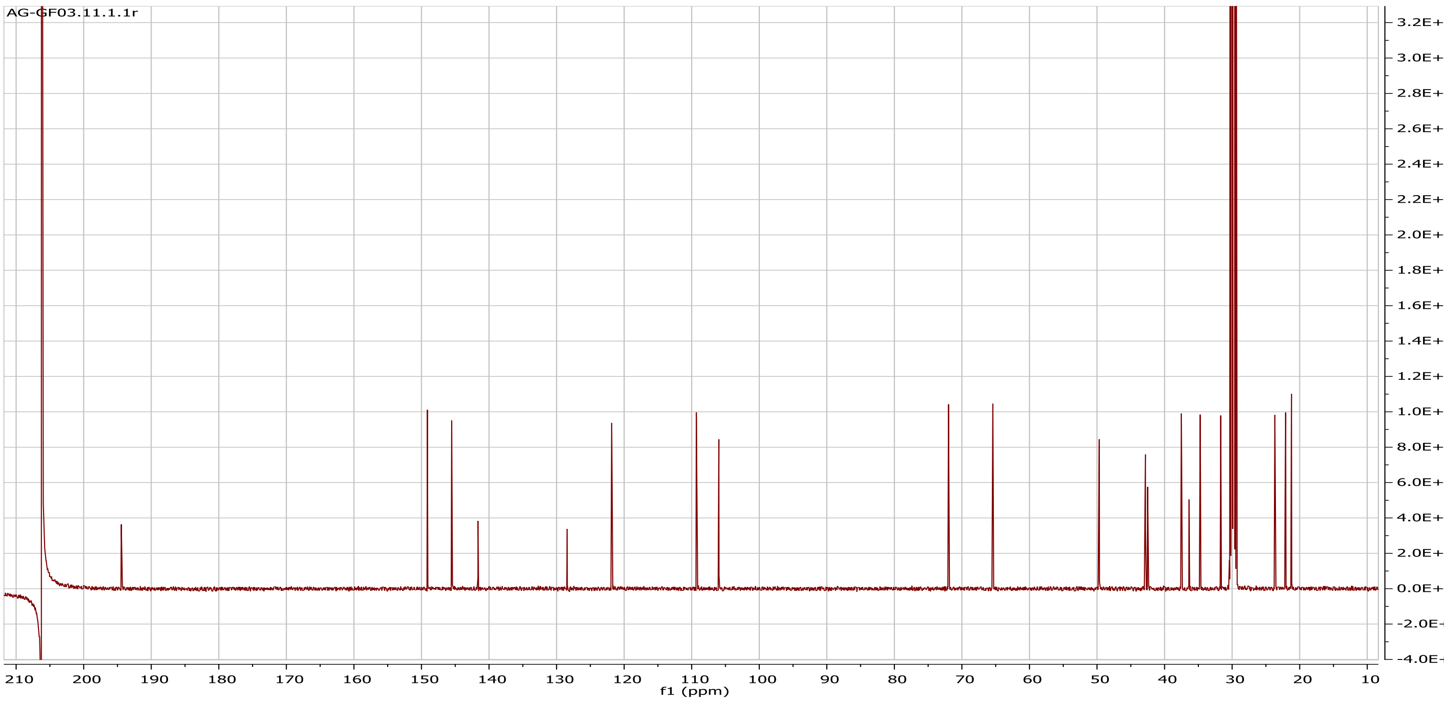

Figure S6: ^{13}C NMR spectrum (150 MHz, acetone-d_6) of compound 3

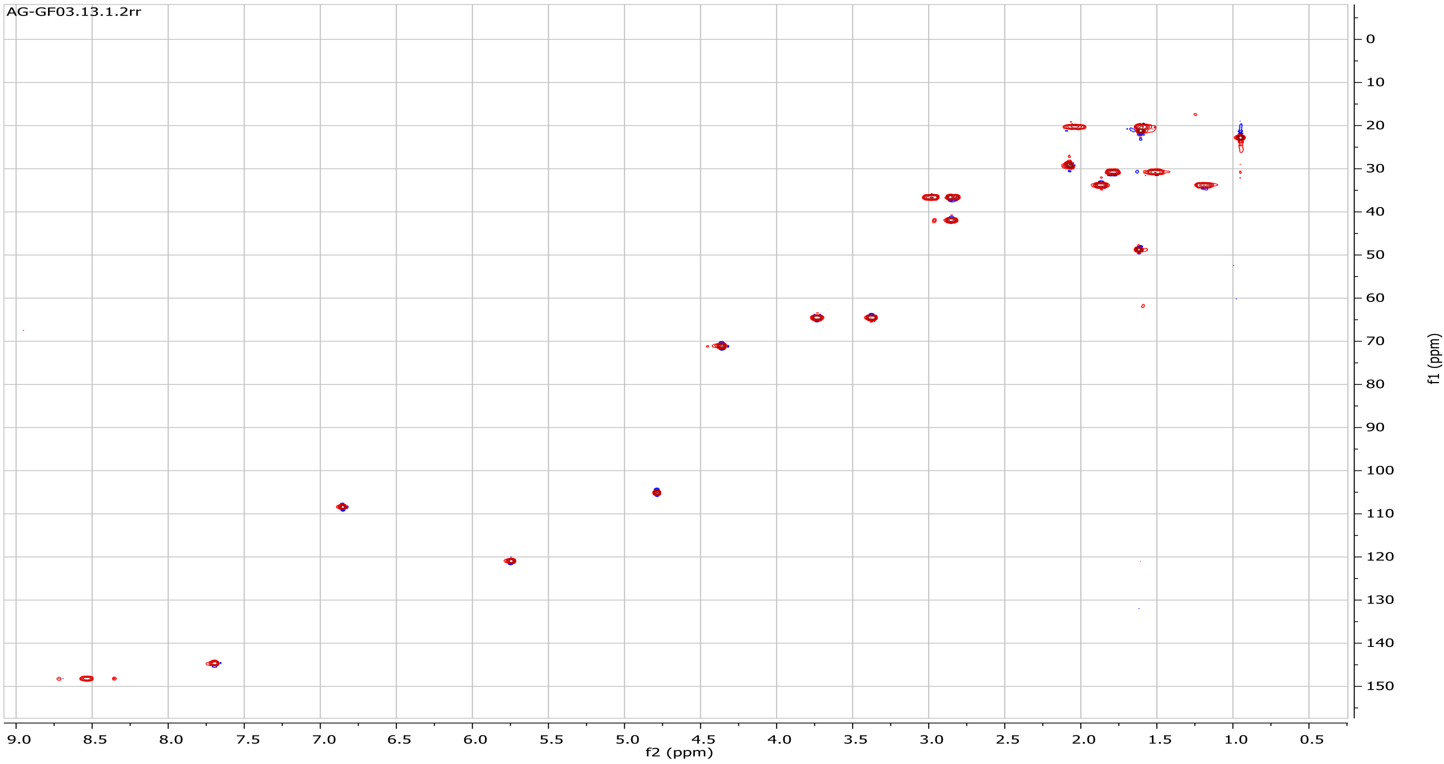

Figure S7: HSQC spectrum of compound 3

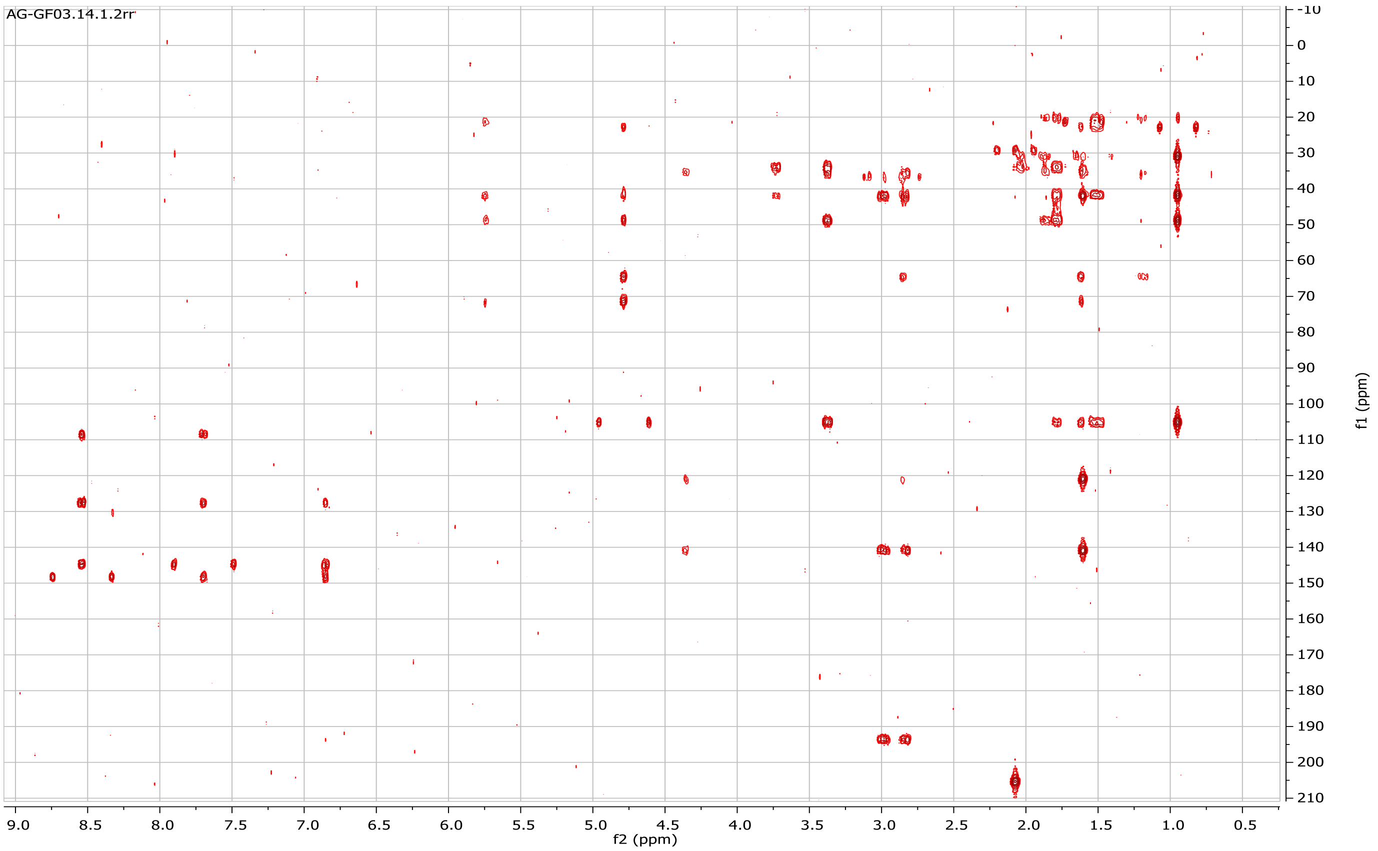

Figure S8: HMBC spectrum of compound 3

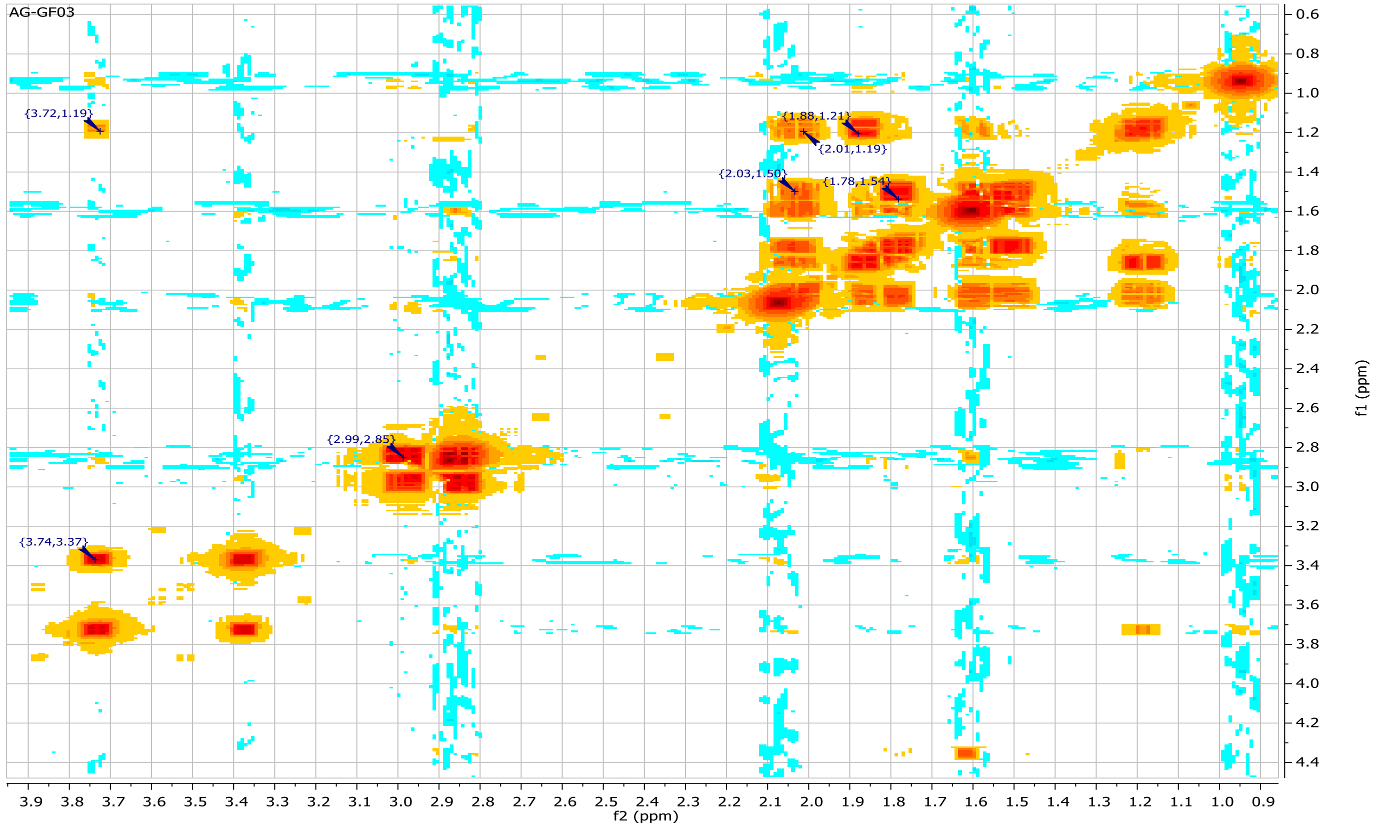

Figure S9: ^{1}H-^{1}H COSY spectrum of compound 3

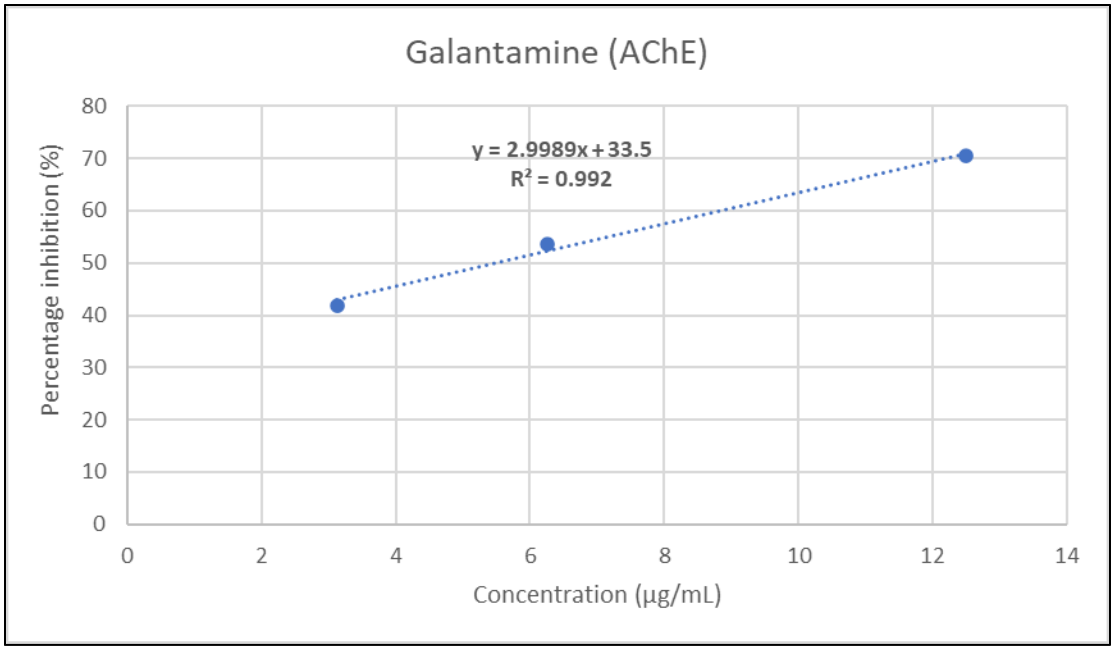

Figure S10: Galantamine graph for IC_{50} calculation of the acetylcholinesterase (AChE) enzyme inhibition (Concentration range 3.125 – 12.5 µg/mL)

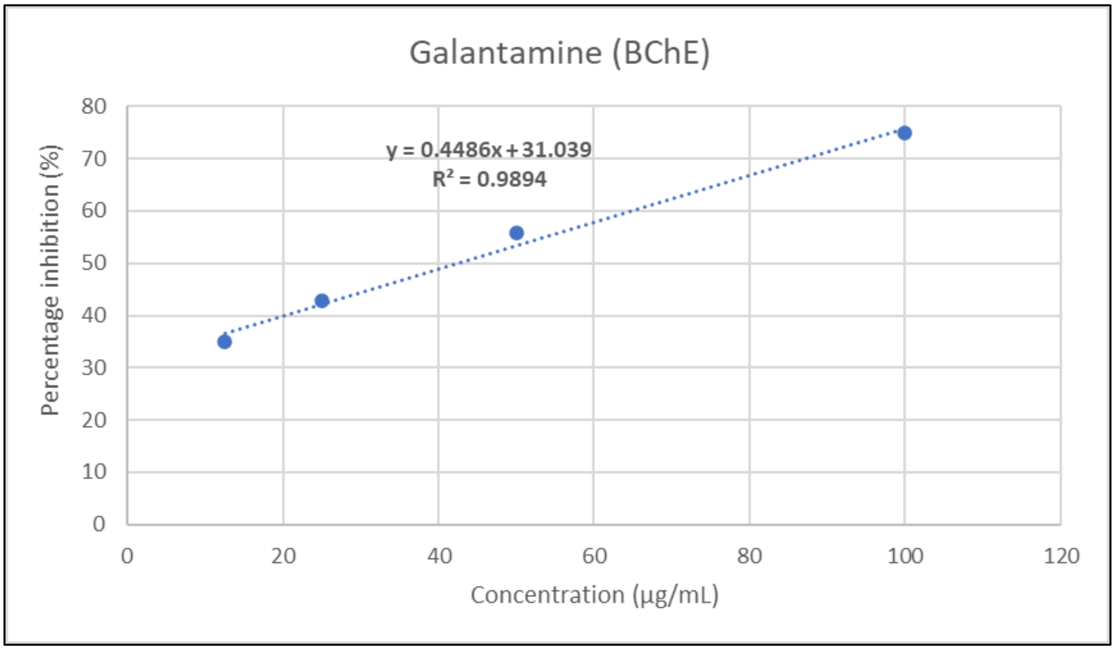

Figure S11: Galantamine graph for IC_{50} calculation of the acetylcholinesterase (AChE) enzyme inhibition (Concentration range 12.5 – 100 µg/mL)

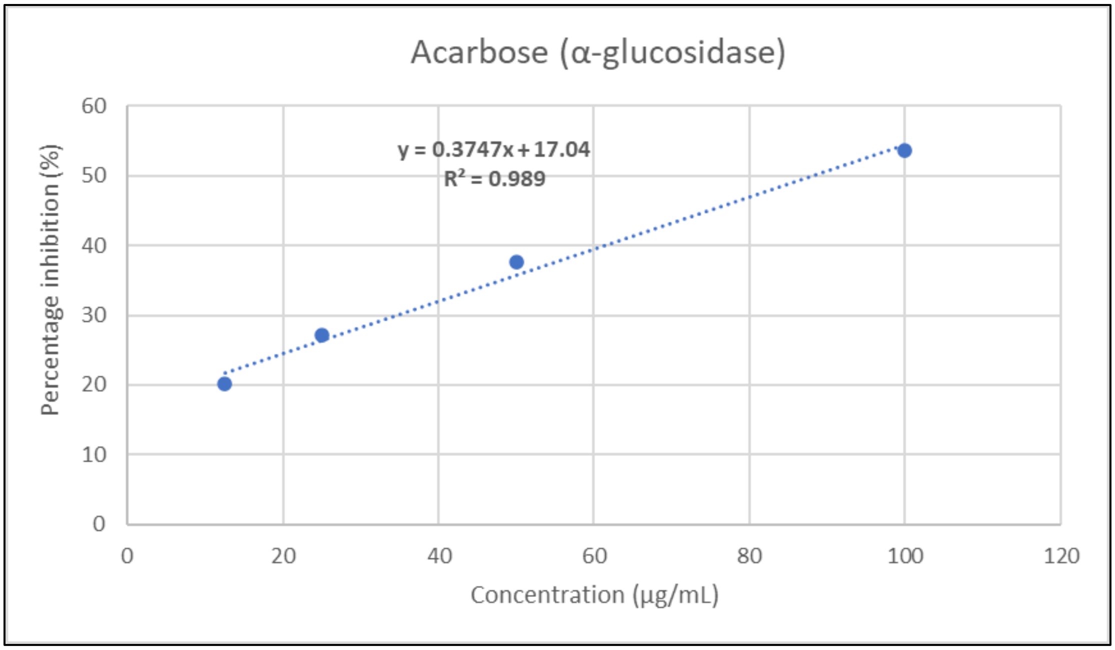

Figure S12: Acarbose graph for IC_{50} calculation of the α-glucosidase enzyme inhibition (Concentration range 12.5 – 100 µg/mL)

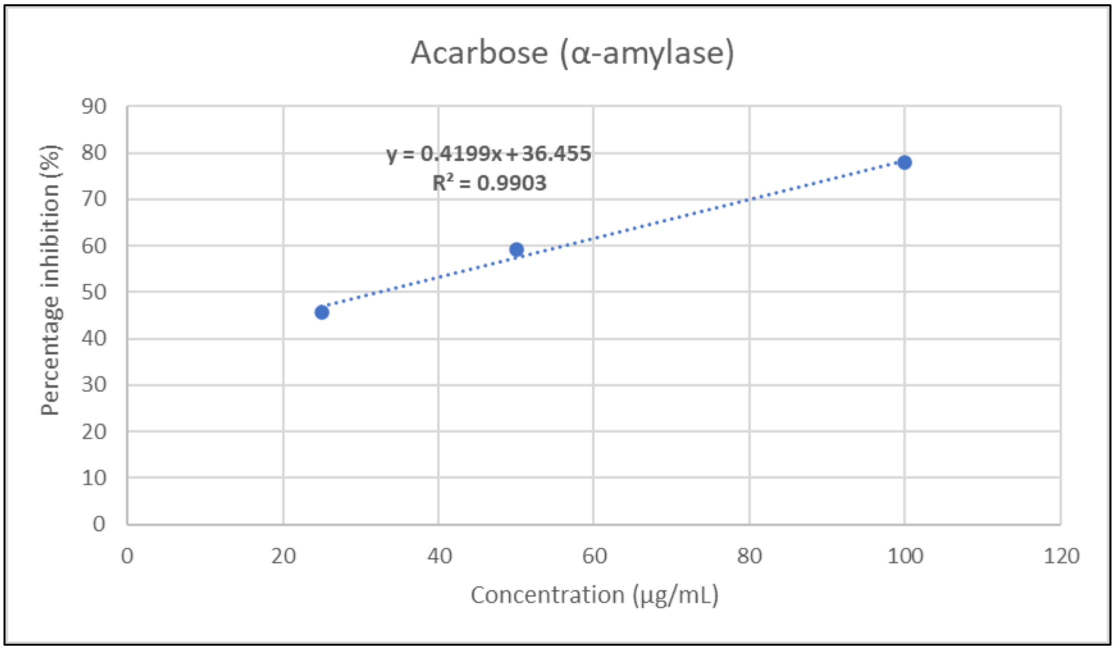

Figure S13: Acarbose graph for IC_{50} calculation of the α-glucosidase enzyme inhibition (Concentration range 25 – 100 µg/mL)

Table S1: Equations used for IC_{50} calculation of the anticholinesterase activity of the samples (Concentration range 25 – 100 μg/mL)

Test sample	Anticholinesterase activity	
	AChE<br>Equation (R^2)	BChE<br>Equation (R^2)
**Leaves extract**	-	y = 0.5394x + 18.6<br>(R^2 = 0.9952)
**Stem extract**	-	y = 0.504x + 16.2<br>(R^2 = 0.9983)
**Hypopurin A**	-	y = 0.4434x + 24.06<br>(R^2 = 0.9906)
**Hypopurin B**	-	y = 0.5049x + 16.15<br>(R^2 = 0.9985)
**Hypopurin E**	-	y = 0.369x + 17.965<br>(R^2 = 0,9877)
**Lupeol**	-	-
**β-Sitosterol glucoside**	-	-
**Stigmasterol glucoside**	-	-
**Stigmasterol & β-sitosterol**	-	-

Table S2: Equations used for IC_{50} calculation of the antidiabetic activity of the samples (Concentration range 25 – 100 μg/mL)

Test sample	Antidiabetic activity	
	α-glucosidase<br>Equation (R^2)	*α*-amylase<br>Equation (R^2)
**Leaves extract**	y = 0.2881x + 36.055<br>(R^2 = 0.9918)	y = 0.3931x + 22.34<br>(R^2 = 0.9895)
**Stem extract**	y = 0.4349x + 30.18<br>(R^2 = 0.9902)	y = 0.4543x + 20.6<br>(R^2 = 0.9878)
**Hypopurin A**	y = 0.4164x + 8.685<br>(R^2 = 0.9997)	y = 0.507x + 19.355<br>(R^2 = 0.9987)
**Hypopurin B**	y = 0.308x + 28.075<br>(R^2 = 0.9996)	y = 0.2837x + 30.25<br>(R^2 = 0.9996)
**Hypopurin E**	y = 0.2576x + 30.325<br>(R^2 = 0.9987)	y = 0.3143x + 26.8<br>R^2 = 0.9912
**Lupeol**	y = 0.4024x + 21.895<br>(R^2 = 0.9985)	-
**β-Sitosterol glucoside**	-	-
**Stigmasterol glucoside**	-	-
**Stigmasterol & β-sitosterol**	-	-
**Acarbose**	y = 0.3747x + 17.04<br>(R^2 = 0.989)	y = 0.4199x + 36.455<br>(R^2 = 0.9903)
